# Ca^2+^-PP2B-PSD-95 axis: A novel regulatory mechanism of the phosphorylation state of Serine 295 of PSD-95

**DOI:** 10.1371/journal.pone.0313441

**Published:** 2024-11-07

**Authors:** Takahiko Chimura, Toshiya Manabe

**Affiliations:** Department of Basic Medical Sciences, Institute of Medical Science, Division of Neuronal Network, University of Tokyo, Tokyo, Japan; Children’s Hospital Affiliated of Zhengzhou University: Zhengzhou Children’s Hospital, CHINA

## Abstract

The phosphorylation state of PSD-95 at Serine 295 (Ser295) is important for the regulation of synaptic plasticity. Although the activation of NMDA receptors (NMDARs), which initiates an intracellular calcium signaling cascade, decreases phosphorylated Ser295 (pS295) of PSD-95, the molecular mechanisms are not fully understood. We found that the calcium-activated protein phosphatase PP2B dephosphorylated pS295 not only in basal conditions but also in NMDAR-activated conditions in cultured neurons. The biochemical assay also revealed the dephosphorylation of pS295 by PP2B, consistently supporting the results obtained using neurons. The newly identified calcium signaling cascade “Ca^2+^-PP2B-PSD-95 axis” would play an important role in the molecular mechanism for NMDA receptor-dependent plasticity.

## Introduction

The excitatory synaptic transmission is virtually mediated by AMPA-type glutamate receptors (AMPARs) and the regulation of their surface expression at postsynaptic sites is crucial not only for basal synaptic transmission but also for synaptic plasticity, such as long-term potentiation (LTP) and long-term depression (LTD) [1–5]. NMDA receptors, glutamate-gated calcium-permeable ion channels, play pivotal roles in triggering synaptic plasticity [2,4,6]. Thus, the activity-dependent influx of calcium ions through NMDA receptors and downstream calcium signaling cascades are expected to affect the machinery that regulates surface expression of AMPARs [6–9]. However, the precise molecular basis remains elusive.

At postsynaptic sites, a wide variety of membrane proteins and signal transduction proteins are organized into a functional macromolecular complex [3,8,10–14]. In this process, PSD-95, a most extensively studied scaffold protein at the postsynaptic sites, plays central roles in assembling the multiple proteins for synaptic transmission [11–16]. For example, PDZ domains of PSD-95 interact with TARPs, an auxiliary subunit of AMPARs, contributing to the surface expression of AMPARs at the postsynaptic membrane [17–19]. It is reported that an increase of the expression of PSD-95 in neurons facilitates surface expression of AMPARs at the synaptic membranes and also enhances synaptic transmission mediated by AMPARs [18,20]. In contrast, decrease of PSD-95 downregulates them [21,22].

The growing body of evidence showing that PSD-95 is a critical regulator for the strength of synaptic transmission through association with AMPARs indicates that identification of the regulatory mechanisms affecting the properties of PSD-95 is an important issue for understanding the molecular basis for synaptic plasticity. To date, several studies have revealed that the localization and mobility of PSD-95 in the postsynaptic site are regulated by the phosphorylation at Serine 295 (Ser295) [23,24], and that c-Jun NH2-terminal kinases (JNKs) phosphorylates this site [23]. It is also shown that this modification regulates the localization of AMPARs at postsynaptic sites in NMDA-triggered LTD [23,24]. Furthermore, it is reported that activation of NMDA receptors drastically decreases the phosphorylated isoform of Ser295 (pS295) [23,24]. Because activation of NMDA receptors triggers an influx of extracellular calcium ions via NMDA receptors, we speculate that dephosphorylation of pS295 of PSD-95 is likely to be calcium-dependent.

PP2B (protein phosphatase 2B, also known as calcineurin), a heterodimer composed of catalytic (PP2B-A) and regulatory (PP2B-B) subunits, is a calcium/calmodulin-activated serine/threonine phosphatase conserved among eukaryotes. When intracellular calcium concentration is increased, calcium-sensing protein calmodulin (CaM) binds to Ca^2+^ ions with its Ca^2+^-binding domain called EF-hand, then Ca^2+^/CaM activates PP2B via binding to PP2B-A subunit [25,26]. It is noteworthy that PP2B plays an important role in synaptic plasticity including NMDA receptor-dependent LTD [26–32]. These results inspired us to hypothesize that the calcium-activated phosphatase PP2B regulates the phosphorylation state at Ser295 of PSD-95. To test this hypothesis, we carefully evaluate the role of PP2B in the regulation of the phosphorylation state at Ser295 of PSD-95.

Here, we show that distinct types of PP2B inhibitors increased pS295 of PSD-95 without apparent JNK activation in cultured mouse cortical neurons. The increased pS295 was also observed when an influx of calcium ions into neurons was inhibited using the calcium chelator EGTA. We also detected the contribution of PP2B during the NMDA-induced decrease of pS295. In addition to these results obtained in neurons, the biochemical analysis revealed that PP2B dephosphorylated endogenous pS295 of PSD-95 in mouse brain extracts. Our results suggest that the novel signaling cascade “Ca^2+^-PP2B-PSD-95 axis” is an important molecular basis for the regulation of synaptic plasticity.

## Results

### Inhibition of PP2B activity increases phospho-S295 of PSD-95 in primary cultures without JNK activation

**Fig 1:**
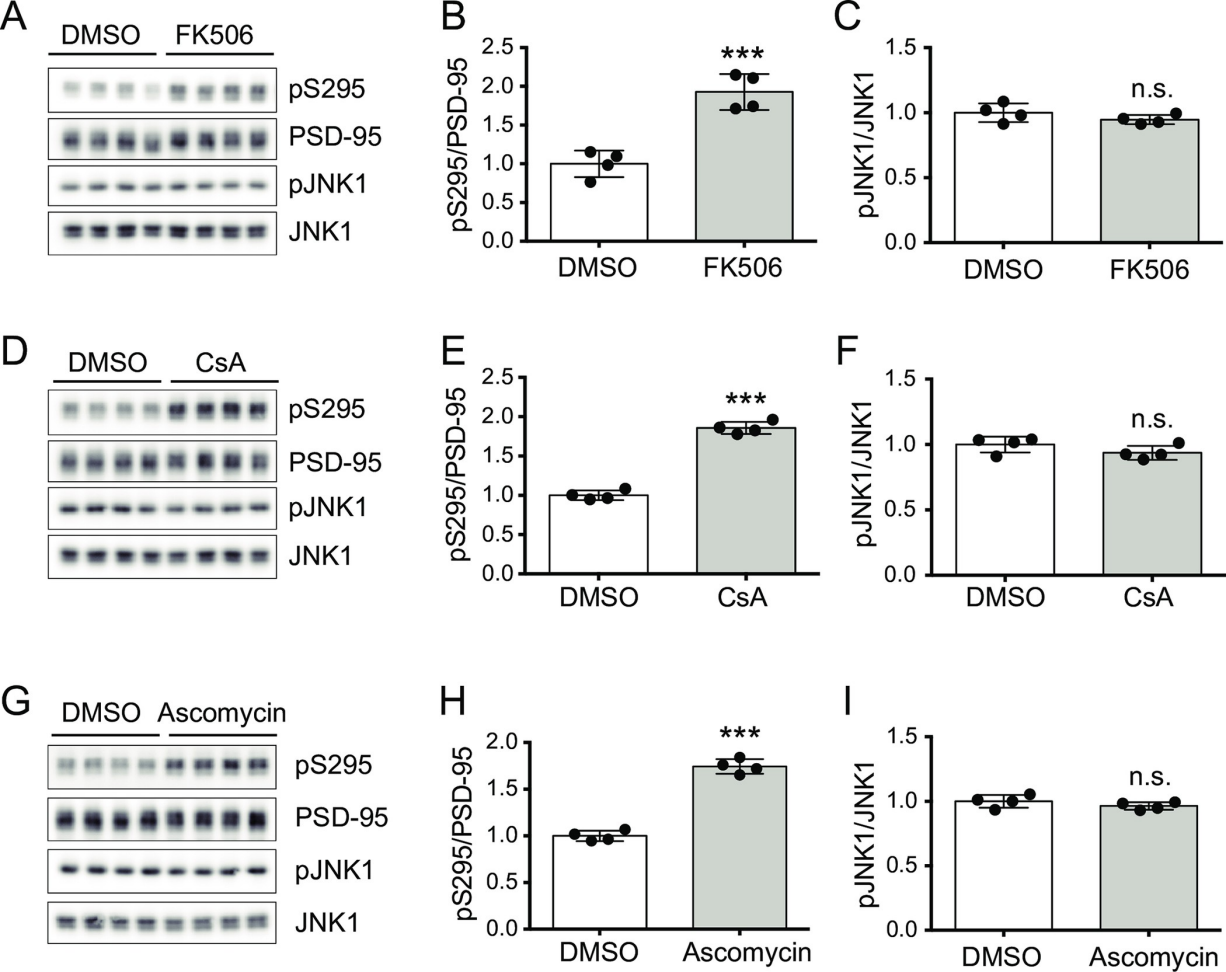
PP2B inhibitors increase phosphorylated Ser295 of PSD-95 in cultured mouse cortical neurons. (A) Western-blot analyses of phosphorylated Ser295 of PSD-95 (pS295), total PSD-95, an active form of JNK1 (p-JNK1) and total JNK1 in untreated (DMSO) and FK506-treated (2 μM, 45 min) primary mouse cortical neurons. (B, C) Quantification of pS295/PSD-95 (B) and pJNK1/JNK1 (C) with the FK506 treatment relative to the untreated condition (DMSO) shown in (A). (D-F) Western-blot analysis (D) and quantification (E, F) of cyclosporin A (CsA)-treated (2 μM, 45 min) neurons. (G-I) Western-blot analysis (G) and quantification (H, I) of ascomycin-treated (2 μM, 45 min) neurons. The data are represented as the mean ± standard deviation overlaid with individual data points (n = 4, each). ***P < 0.001 by the unpaired Student’s *t*-test; n.s., not significant.

In order to test whether PP2B regulates the phosphorylation state at Ser295 of PSD-95, mouse cortical neurons were treated with FK506, a commonly used PP2B inhibitor, and phospho-S295 of PSD-95 (pS295) was measured by Western-blot analyses. Because it is known that JNK phosphorylates S295 of PSD-95, we also measured an active form of JNK by detecting phospho-JNK (pJNK). As shown in Fig 1A–1C, the pS295 signal was apparently increased by the FK506 treatment without JNK activation. Similar results were observed using other PP2B inhibitors, cyclosporin A (CsA) (Fig 1D–1F) and FK506 analog ascomycin (Fig 1G–1I), suggesting that PP2B dephosphorylates S295 of PSD-95.

Because PP1 and PP2A are also suggested to be involved in the dephosphorylation of S295 of PSD-95 [23], we tested whether this is the case in our primary cultures. As shown in S1 Fig, pS295 of PSD-95 was increased by the PP1/PP2A inhibitor calyculin A where JNK activation concomitantly occurred, suggesting that PP1/PP2A could indirectly regulate pS295 by regulating activities of JNK.

### Calcium influx causes PP2B-mediated dephosphorylation of pS295 of PSD-95

**Fig 2:**
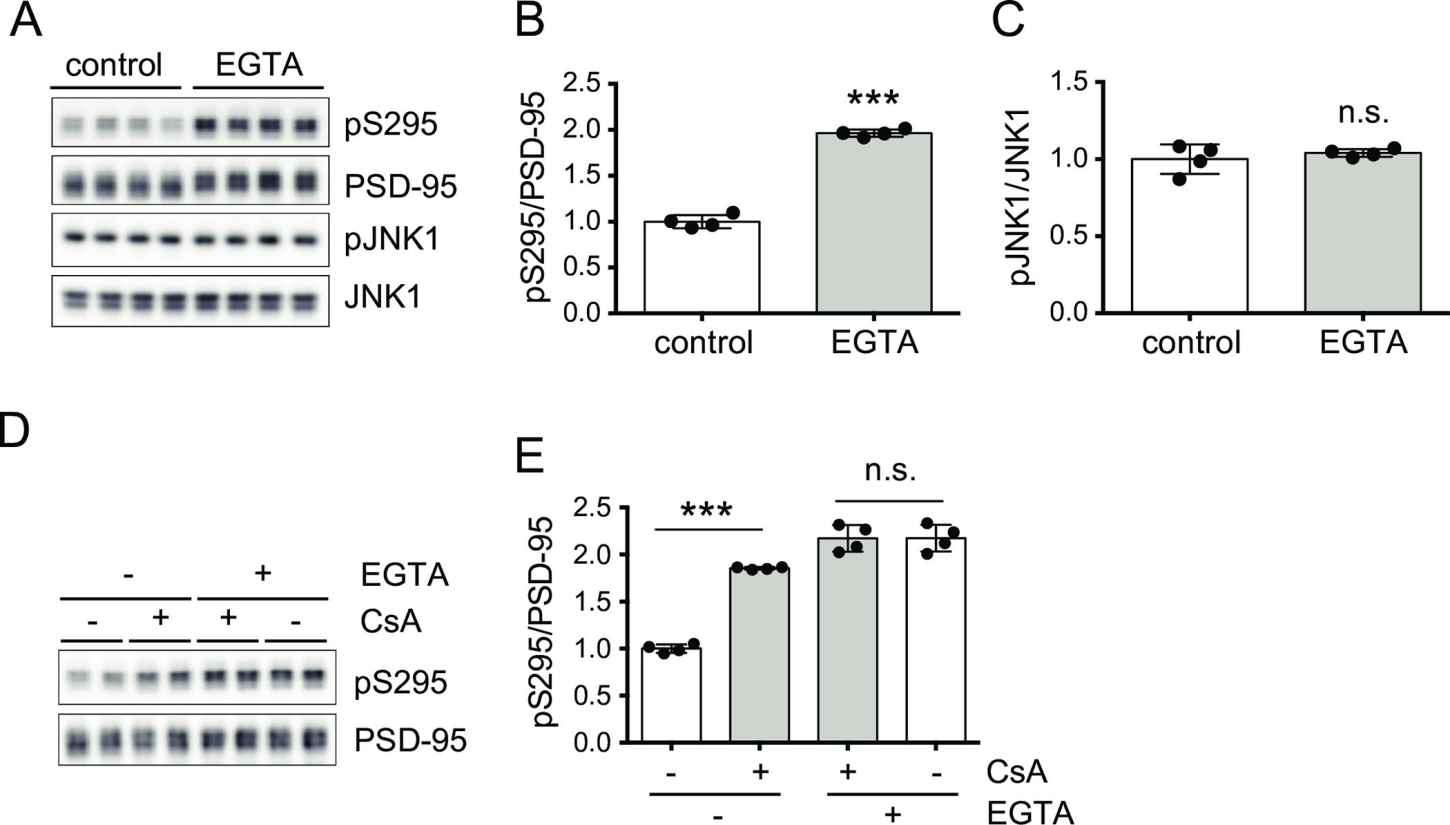
Influx of extracellular calcium activates PP2B. (A) Western-blot analyses of phosphorylated Ser295 of PSD-95 (pS295), total PSD-95, an active form of JNK1 (pJNK1) and total JNK1 in untreated (control) and EGTA-treated (2.5 mM, 1 h) primary mouse cortical neurons. (B, C) Quantification of pS295/PSD-95 (B) and pJNK1/JNK1 (C) with the EGTA treatment relative to the untreated condition (control) shown in (A). The data are represented as the mean ± standard deviation overlaid with individual data points (n = 4). ***P < 0.001 by the unpaired Student’s *t*-test; n.s., not significant. (D, E) Pretreatment by EGTA (2.5 mM, 45 min) before CsA treatment (2 μM, 45 min) occluded the effect of CsA. The data are represented as the mean ± standard deviation overlaid with individual data points (n = 4 from two independent experiments). ***P < 0.001 by two-way ANOVA with the post-hoc Tukey’s multiple comparison test; n.s., not significant. *F*(1,12) = 68.97, *P* < 0.0001 for EGTA, *F*(1,12) = 211.5, *P* < 0.0001 for CsA, and *F*(1,12) = 69.50, *P* < 0.0001 for interaction.

If PP2B truly dephosphorylates pS295 of PSD-95 *in vivo*, it is expected that the inhibition of calcium influx would also increase pS295 of PSD-95, because PP2B is a calcium-dependent protein phosphatase. To test this possibility, the effect of chelation of extracellular calcium by EGTA on pS295 of PSD-95 was examined. As shown in Fig 2A–2C, EGTA treatment increased pS295 without JNK activation, strongly suggesting that calcium influx induces dephosphorylation of pS295 of PSD-95 via PP2B activation.

If calcium influx causes PP2B-mediated dephosphorylation of pS295 of PSD-95, it is expected that chelation of extracellular calcium occludes the effect of PP2B inhibition by the PP2B inhibitor. As shown in Fig 2D and 2E, PP2B inhibition by CsA did not increase pS295 of PSD-95 in the presence of EGTA. Thus, the chelation of extracellular calcium by EGTA occludes the effect of PP2B inhibition, indicating that calcium influx induces PP2B-mediated dephosphorylation of pS295 of PSD-95.

### PP2B is involved in NMDA-induced dephosphorylation of pS295 of PSD-95

**Fig 3:**
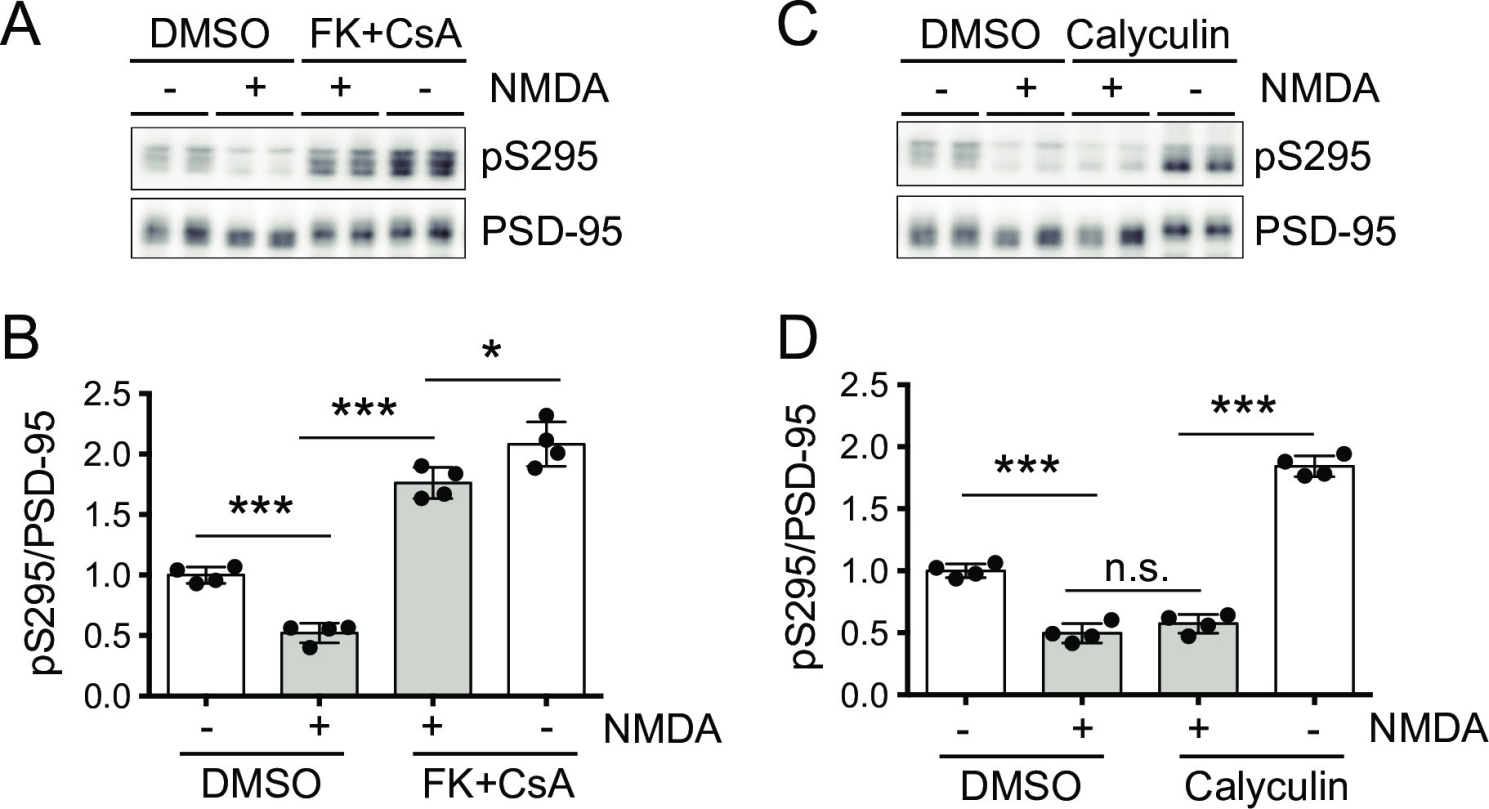
PP2B, but not PP1/PP2A, is involved in the NMDA-induced dephosphorylation of pS295. (A, B) Western-blot analysis (A) and quantification (B) showing that pretreatment of the mixture of PP2B inhibitors (FK506 and CsA) suppressed dephosphorylation of pS295 induced by the NMDA treatment (20 μM, 15 min) in primary mouse cortical neurons. The data are represented as the mean ± standard deviation overlaid with individual data points (n = 4 from two independent experiments). *P < 0.05, ***P < 0.001 by two-way ANOVA with the post-hoc Tukey’s multiple comparison test. *F*(1,12) = 41.36, *P* < 0.0001 for FK+CsA, *F*(1,12) = 349.9, *P* < 0.0001 for NMDA, and *F*(1,12) = 1.611, *P* = 0.2285 for interaction. (C, D) Western-blot analysis (C) and quantification (D) showing that pretreatment of the PP1/PP2A inhibitor calyculin A had no apparent suppressive effect. The data are represented as the mean ± standard deviation overlaid with individual data points (n = 4 from two independent experiments). ***P < 0.001 by two-way ANOVA with the post-hoc Tukey’s multiple comparison test; n.s., not significant. *F*(1,12) = 569.1, *P* < 0.0001 for Calyculin, *F*(1,12) = 153.3, *P* < 0.0001 for NMDA, and *F*(1,12) = 106.0, *P* < 0.0001 for interaction.

It is reported that repression of NMDA-type glutamate receptors (NMDARs) using their antagonist D-APV increases pS295 of PSD-95, whereas the activation of NMDARs using their agonist NMDA decreases pS295 of PSD-95 [23,24], indicating that NMDARs play a pivotal role in the regulation of pS295 of PSD-95. Taking into consideration that activation of NMDARs induces calcium influx and that PP2B is a Ca^2+^-activated protein phosphatase, it is quite rational to hypothesize that the NMDA-induced decrease of pS295 of PSD-95 is mediated by PP2B. To test this hypothesis, primary neuronal cultures were pretreated with PP2B inhibitors followed by NMDA treatment and examined whether the PP2B inhibitors suppressed NMDA-induced dephosphorylation of pS295 of PSD-95 (Fig 3). To suppress the PP2B activity as much as possible, a mixture of FK506 and CsA was used in this experiment, because FK506 and CsA bind to distinct cellular cofactors FKBPs and cyclophilins, respectively for the inhibition of PP2B activity [33,34]. In the control condition (i.e. without PP2B inhibitors), NMDA treatment (20 μM, 15 min) reduced pS295 of PSD-95 as previously reported [23,24]. When pretreated with PP2B inhibitors (FK506 and CsA, 5 μM each, 45min), NMDA-induced dephosphorylation of pS295 of PSD-95 was apparently suppressed, and the phosphorylation of pS295 was still maintained at higher levels than that in untreated conditions. These results indicate that PP2B is involved in NMDA-induced dephosphorylation of pS295. In contrast, the PP1/PP2A inhibitor calyculin A did not suppress the reaction at all (Fig 3C and 3D). These results indicate that PP2B is a major phosphatase in NMDA-induced dephosphorylation of pS295.

### PP2B dephosphorylates pS295 *in vitro*

**Fig 4:**
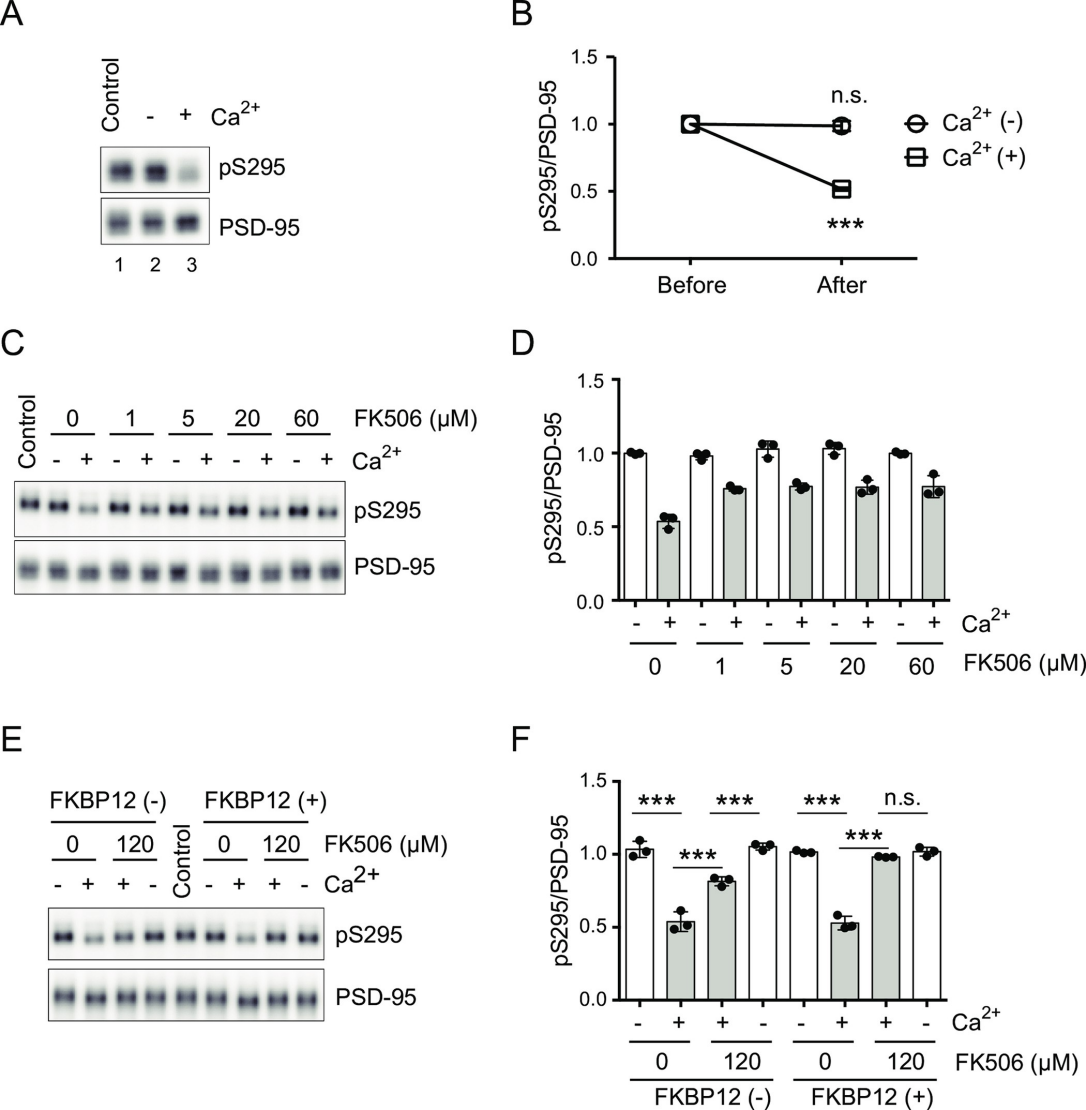
PP2B dephosphorylates pS295 *in vitro*. (A) Western-blot analysis of the *in-vitro* dephosphorylation assay using mouse brain extracts. The “Control” sample (lane 1) was not subject to the dephosphorylation assay. (B) Quantification of dephosphorylation *in vitro* (pS295/PSD-95) incubated with calcium (Ca^2+^(+)) or without calcium (Ca^2+^(-)), relative to no-incubation (Control) conditions. The data are represented as the mean ± standard deviation (n = 3 from three independent experiments). ***P < 0.001 by the unpaired Student’s *t*-test; n.s., not significant. (C, D) Western-blot analysis (C) and quantification (D) of the efficiency of suppression by different doses of FK506 on the Ca^2+^-dependent dephosphorylation of pS295 *in vitro*. (E, F) Western-blot analysis (E) and quantification (F) showing that coapplication of FK506 and FKBP12 efficiently suppressed the Ca^2+^-dependent dephosphorylation of pS295 *in vitro*. The data are represented as the mean ± standard deviation overlaid with individual data points (n = 3 from three independent experiments). ***P < 0.001 by two-way ANOVA with the post-hoc Tukey’s multiple comparison test; n.s., not significant. *F*(1,16) = 383.3, *P* < 0.0001 for FKBP12+FK506, *F*(3,16) = 48.44, *P* < 0.0001 for Ca^2+^, and *F*(3,16) = 47.06, *P* < 0.0001 for interaction.

In order to solidify our *in-vivo* findings that PP2B dephosphorylates pS295 of PSD-95, we developed an *in-vitro* assay system for the detection of dephosphorylation of pS295. When the extracts of the mouse brain were incubated in the reaction buffer without calcium, the level of pS295 (Fig 4A, lane 2) was the same as the control (i.e., without incubation: Fig 4A, lane 1). On the other hand, incubations with calcium apparently decreased the level of pS295 (Fig 4A, lane 3). Thus, we successfully detected the dephosphorylation of pS295 of PSD-95 in a calcium-dependent manner, using crude mouse brain extracts (Fig 4A and 4B).

Notably, in our *in-vitro* dephosphorylation assay, phosphatases other than PP2B were active in “without Ca^2+^” conditions (S2 Fig). In this condition, pS295 of PSD-95 did not change after incubation (Figs 4A, 4B and S2A), whereas the level of phosphorylation of Serine 9 of GSK3β (pGSK3β) was reduced, which was completely inhibited by the PP1/PP2A inhibitor calyculin A. This result indicates that active PP1/PP2A dephosphorylated Serine 9 of GSK3, which is consistent with the previous study that Serine 9 of GSK3β is a target of PP1/PP2A [35]. This result also indicates that pS295 is not a preferential target of PP1/PP2A. Thus, the decrease of pS295 observed in our *in-vitro* assay is highly specific.

Next, in order to confirm that the decrease of pS295 depends on PP2B activity, we examined whether the PP2B inhibitor FK506 suppressed the dephosphorylation of pS295 (Fig 4C and 4D). To our surprise, FK506 suppressed the dephosphorylation only partially. The efficiency of suppression already reached a plateau at 1 μM and was not improved even with higher doses of FK506. Taking into account the fact that FK506 requires endogenous FKBPs (FK506-binding proteins) as cofactors to inhibit PP2B [33,34], the amount of FKBPs in the brain extracts might be the limiting factor for the PP2B inhibition. If this is true, exogenously supplied recombinant FKBPs are expected to improve the efficiency of suppression by FK506. Thus, we examined whether exogenously supplied recombinant FKBP12 together with excessive doses of FK506 could suppress the calcium-dependent dephosphorylation of pS295. As shown in Fig 4E and 4F, dephosphorylation of pS295 of PSD-95 was suppressed highly efficiently in the presence of both recombinant FKBP12 and FK506, compared with that in the “FK506 only” condition. These results indicate that PP2B plays major roles in the dephosphorylation of pS295 of PSD-95 in our *in-vitro* assay system, and that partial suppression of the dephosphorylation in the “FK506 only” condition is caused by the insufficient amount of endogenous FKBPs in the brain extract.

We also tested the efficiency of the suppression of dephosphorylation by CsA, which requires endogenous proteins cyclophilins as cofactors to inhibit PP2B [33]. As shown in S3A and S3B Fig, however, CsA suppressed the dephosphorylation only partially, indicating the possibility that the amount of cyclophilins in the brain extract was also insufficient for full inhibition of PP2B. As shown in S3C and S3D Fig, the efficiency of suppression by the mixture of excessive amount of FK506 and CsA was still incomplete, suggesting that the amount of endogenous FKBPs and cyclophilins was insufficient for the complete inhibition of PP2B with the mixture of FK506 and CsA.

## Discussion

### Identification of the “Ca^2+^-PP2B-PSD-95 axis”

The phosphorylation state of S295 of PSD95 is associated with the stability of PSD-95 at the postsynaptic sites and the targeting of AMPARs at the synaptic membrane [23,24]. In this study, we revealed that S295 of PSD-95 was a novel target for PP2B. One of our results supporting this conclusion is that the treatments with PP2B inhibitors elicited the increase of pS295 in cultured neurons without the activation of JNK1, a responsible kinase for the phosphorylation of S295 of PSD-95 (Fig 1). The previous study suggests that the involvement of PP2B in the regulation of pS295 is unlikely based on the results that the PP2B inhibitor ascomycin does not induce the increase of pS295 in neuronal cultures [23]. In our cases, however, an increase of pS295 is observed not only with ascomycin, but also with the commonly used PP2B inhibitor FK506 or CsA. These results strongly suggest that PP2B is active in our basal culture conditions. In our experiments, mouse cortical neurons cultured in Neurobasal medium supplemented with B-27 and glutamine were treated with ascomycin (2 μM, 45 min) at DIV 19–21 (see also Materials and methods). In the previous experiments, on the other hand, rat hippocampal neurons cultured in MEM with fetal calf serum (FCS) and several supplements were treated with ascomycin (2 μM, 2 hr) at DIV 25–28 [23,36,37]. The differences of the experimental conditions, such as species, brain regions, cell culture media, culture period, and treatment duration might cause this inconsistency.

The PP1/PP2A inhibitor calyculin A induced the increase of pS295 (S1 Fig) as observed in the previous study [23]. Notably, however, JNK1 activation concomitantly occurred with the increase of pS295 (S1 Fig), which is consistent with the report that JNK1 is repressed by PP1 [38]. Furthermore, okadaic acid, another type of PP1/PP2A inhibitor, activates JNK1 and induces the increase of pS295 of PSD-95 [39,40]. These results suggest that the increase of pS295 caused by PP1/PP2A inhibition could be mediated by activated JNK1. Together with the *in-vitro* study showing that pS295 was dephosphorylated by PP2B (Fig 4E and 4F), our study identified the novel calcium signaling cascade “Ca^2+^-PP2B-PSD-95 axis” regulating the phosphorylation state of PSD-95.

It is expected that the increase of pS295 of PSD-95 by FK506 (and other PP2B inhibitors) (Fig 1) could increase surface expressions of AMPARs and the synaptic potentiation, because the phosphorylation at Ser295 of PSD-95 is shown to promote these cellular reactions [23]. It is of note that several reports support this expectation. In cultured primary neurons, for example, FK506 treatment increases the surface expressions of AMPARs [41,42]. Experiments using acute brain slices revealed that synaptic potentiation is induced when FK506 is postsynaptically applied to hippocampal CA1 neurons [43–45]. Thus, we believe that the “Ca^2+^-PP2B-PSD-95 axis” will provide novel insights for understanding the molecular mechanisms of synaptic plasticity.

How does PP2B recognize PSD-95 as its substrate? Because “PP2B-binding motifs” such as LxVP- and PxIxIT-motif are not identified in PSD-95 [46], indirect bindings between PP2B and PSD-95, rather than direct ones, might be more likely. A certain kind of proteins binding to both PP2B and PSD-95, such as AKAP150/79 [47–52] and DLGAP1 [53,54], might facilitate the recognition of PSD-95 by PP2B as mediators of the “Ca^2+^-PP2B-PSD-95 axis”. In future studies, it is also important to determine the PP2B isozyme that dephosphorylates pS295 of PSD-95. PP2B is a heterodimer composed of catalytic (PP2B-A) and regulatory (PP2B-B) subunits. PP2B-A isoforms, α, β1, β2 and γ, are encoded by three genes: Ppp3ca, Ppp3cb (alternatively spliced to generate β1 and β2) and Ppp3cc [55,56]. PP2B-B is encoded by Ppp3r1 and Ppp3r2. The four genes other than testis-specific isotype Ppp3r2 are expressed in central nervous systems. Taking that PP2B isozymes that contain different PP2B-A isoforms show distinct substrate selectivity and intracellular localization [57,58], it is a critical issue to determine isotypes of PP2B responsible for the dephosphorylation pS295 of PSD-95, by knockdown, knockout, or overexpression of each PP2B-A subunit.

### Putative roles of the “Ca^2+^-PP2B-PSD-95 axis” in LTD

It is reported that activation of NMDARs, which is required for LTD induction, promotes dephosphorylation of pS295 of PSD-95 (Fig 3 in this study; Kim et al., 2007 [23]), and that phospho-mimic mutation of S295 of PSD-95 (S295D) inhibits LTD induction [23], indicating that dephosphorylation of S295 of PSD-95 is required for LTD induction. Meanwhile, it is also known that PP2B is required for NMDA receptor-dependent LTD, whose induction is inhibited by FK506 or CsA [26–32]. Although the involvement of both S295 of PSD-95 and PP2B in NMDAR-dependent LTD has been indicated, the detailed molecular mechanisms underlying LTD have been unclear. In this study, we identified simple relationships between them: substrate and enzyme. As shown in Fig 3, inhibition of PP2B attenuates dephosphorylation of pS295 of PSD-95 by NMDA treatment and pS295 levels remain high, which is expected to result in the loss of LTD based on the previous report [23].

In the experiment shown in Fig 3, we noticed that coapplication of FK506 and CsA (5 μM, each) did not completely suppress the decrease of pS295 by NMDA treatment. We also examined whether increased amount of PP2B inhibitors improved the efficiency of the suppression, but no improvement was observed using twice the amount of inhibitors (i.e., FK506 and cyclosporine A, 10 μM each) (S4 Fig). These results seem to be consistent with our *in-vitro* results (S3 Fig) showing that PP2B cannot be completely suppressed by coapplication of a high enough dose of FK506 and CsA due to the insufficient amount of their endogenous cofactors FKBPs and cyclophilins. To achieve maximal efficiency of PP2B suppression *in vivo*, exogenously expressed FKBPs or cyclophilins might be required just as is the case *in vitro* (Fig 4E and 4F).

### Putative roles of the “Ca^2+^-PP2B-PSD-95 axis” in neurological side effects of immunosuppressive therapy

Both FK506 (also known as tacrolimus) and cyclosporin A are clinically used immunosuppressive drugs [59]. In T-cells, dephosphorylation of nuclear factor of activated T-cell family of transcription factors (NFATc1-c4) by PP2B is required for their translocation into the nucleus and then triggers immune responses [60,61]. FK506 and cyclosporin A exhibit immunosuppressive effects by inhibition of the dephosphorylation of NFATs [60,61]. While the inhibition of PP2B is effective in preventing graft rejection, immunosuppressive therapy using PP2B inhibitors can cause neurotoxic side effects such as headache, seizure, and memory impairment [62–66]. To reduce these side effects of immunosuppressants, understanding the molecular basis via the identification of substrates of PP2B in the central nervous system is an important issue. Taking into account that PSD-95 plays central roles in the regulation of synaptic functions via interacting multiple proteins [3,15], the dysfunction of its regulation by PP2B potentially causes a broad range of neurological symptoms. Furthermore, unidentified substrates of PP2B in the central nervous system might also be involved in the neurological side effects caused by immunosuppressive therapy. The *in-vitro* dephosphorylation assay developed in this study will be useful for the screening of novel substrates of PP2B, as well as for the screening of immunosuppressive compounds that minimally affect PSD-95 phosphorylation.

## Materials and methods

### Reagents

NMDA was obtained from Sigma-Aldrich (St. Louis, MO, USA), EGTA from Wako Chemicals (Osaka, Japan), FK506 from ChemScene (Monmouth Junction, NJ, USA), ascomycin from Cayman Chemical (Ann Arbor, MI, USA), cyclosporine A from Nacalai Tesque (Kyoto, Japan), calyculin A from Santa Cruz Biotechnology (Santa Cruz, CA, USA) and recombinant FKBP12 from R&D Systems (Minneapolis, MN, USA). NMDA and EGTA were dissolved in distilled water. FK506, ascomycin, cyclosporine A and calyculin A were dissolved in dimethyl sulfoxide (DMSO).

### Antibodies

The anti-PSD-95 antibody (#MA1-046) was obtained from Thermo Fisher Scientific (Asheville, NC, USA), the anti-phospho-PSD-95 (Ser295) antibody (#45737S), anti-phospho-JNK1 antibody (#9255S) and anti-JNK1 antibody (#3708S) from Cell Signaling Technology (Danvers, MA, USA), and the anti-GSK3β antibody (#sc-81462) and anti-phospho-GSK3β (S9) antibody (#sc-373800) from Santa Cruz Biotechnology.

### Animals and ethics statement

Mice were housed under pathogen-free conditions in the experimental animal facility at Institute of Medical Science, University of Tokyo. All surgery was performed under isoflurane anesthesia, and all efforts were made to minimize suffering. Experiments using mice were approved by the Animal Care and Use Committee of University of Tokyo (Approval Number: PA22-12). The animals were handled in strict accordance with the ARRIVE guidelines and the guidelines of the Animal Care and Use Committee of the University of Tokyo.

### Primary neuronal cultures

Cortical neurons were prepared from C57BL/6J mice at embryonic day 16 (Japan SLC Inc., Shizuoka, Japan) as previously described [67]. Pregnant dams were euthanized using an overdose of isoflurane followed by cervical dislocation, and embryos were dissected out of the uterus. After decapitation of embryos, cortices were harvested in ice-cold HBSS (Nacalai Tesque) and dissociated with 0.25% trypsin-EDTA (Thermo Fisher Scientific) for 20 min at 37°C. The cells were plated onto poly-L-lysine-coated 24-well plates at a density of 2.5 × 10^5^ cells/well in neuron culture medium (Neurobasal (Thermo Fisher Scientific) supplemented with B27 (Thermo Fisher Scientific) and L-glutamine (Thermo Fisher Scientific)). Cultures at 19–21 days *in vitro* (DIV) were used for drug treatments.

### Protein extracts and Western-blot analyses

Proteins were extracted from primary cultures in 24-well plates using Laemmli sample buffer. Clarity ECL Western Substrate (Bio-Rad Laboratories, Hercules, CA, USA) was used for antibody detection, and EZ Capture MG (ATTO, Tokyo, Japan) was used for image capture. Densitometric analyses of bands were performed using ImageJ analysis software. The quantitative data and the original blots for images are included in the Supporting information (S1 Data and S1 Raw images, respectively).

### *In-vitro* dephosphorylation assay

Brain extracts were prepared from adult male C57BL/6J mice (2–3 months old). After euthanasia using an overdose of isoflurane followed by decapitation, the cerebral cortex was excised and homogenized in Lysis buffer (100 mM NaCl, 1 mM EDTA, 20 mM Hepes-NaOH (pH 7.4), 1% (v/v) NP40, 1% (w/v) deoxycholate, 0.1% (w/v) SDS and the protease-inhibitor cocktail (Complete Mini, EDTA-free, Roche Diagnostics, Basel, Switzerland)). The lysates were centrifuged to remove the insoluble matter, and the supernatant was stored at -80°C until use. Sixty micrograms of mouse cortical extracts were diluted in 50 μL of dephosphorylation buffer (100 mM NaCl, 50 mM Tris-HCl (pH 7.5), 0.1 mM EDTA, 1 mM EGTA, 0.01% (v/v) Brij35, 2 mM DTT and the protease-inhibitor cocktail) with or without 1.5 mM CaCl_2_ and incubated at 30°C for 15 min. The dephosphorylation reaction was terminated by adding 50 μL of 2× Laemmli sample buffer, followed by boiling.

### Statistics

All statistical tests were performed using GraphPad Prism 6 software (GraphPad, San Diego, CA, USA).

## Supporting information

S1 Fig The PP1/PP2A inhibitor calyculin A increases phosphorylated Ser295 of PSD-95 concomitantly with the activation of JNK1. (A) Western-blot analyses of phosphorylated Ser295 of PSD-95 (pS295), total PSD-95, an active form of JNK1 (pJNK1) and total JNK1 in untreated (DMSO) and calyculin A-treated (20 nM, 45 min) primary mouse cortical neurons. (B, C) Quantification of pS295/PSD-95 (B) and pJNK1/JNK1 (C) with the FK506 treatment relative to the untreated condition (DMSO) shown in (A). The data are represented as the mean ± standard deviation overlaid with individual data points (n = 4). ***P < 0.001, **P < 0.01 by the unpaired Student’s *t*-test; n.s., not significant.
(TIF)

S2 Fig PP1/PP2A does not dephosphorylate pS295 *in vitro*. (A) Western-blot analysis showing that PP1/PP2A was active in our *in-vitro* dephosphorylation assay. The decrease of phosphorylated GSK3β (pGSK3β(S9)) was completely suppressed by the PP1/PP2A inhibitor calyculin A. (B, C) Quantification of pS295/PSD-95 (B) and pGSK3/β/GSK3β (C) incubated with or without calyculin A relative to the no-incubation (Control) condition. The data are represented as the mean ± standard deviation (n = 3 from three independent experiments). ***P < 0.001 by the unpaired Student’s *t*-test; n.s., not significant.
(TIF)

S3 Fig Coapplication of FK506 and cyclosporine A shows incomplete suppression of pS295 dephosphorylation. (A, B) Western-blot analysis (A) and quantification (B) of the efficiency of suppression by different doses of CsA on the Ca^2+^-dependent dephosphorylation of pS295 *in vitro*. Bars show the mean value and individual data points are shown as black dots (n = 2 from two independent experiments). (C, D) Western-blot analysis (C) and quantification (D) showing the incomplete suppression of the Ca^2+^-dependent dephosphorylation of pS295 *in vitro* by coapplication of FK506 and CsA. The data are represented as the mean ± standard deviation overlaid with individual data points (n = 3 from three independent experiments). **P < 0.01, *P < 0.05 by the unpaired Student’s *t*-test.
(TIF)

S4 Fig Increased dose of PP2B inhibitors does not improve the suppression efficiency of PP2B in the NMDA-induced dephosphorylation of pS295. (A, B) Western-blot analysis (A) and quantification (B) showing that an increased dose of the mixture of PP2B inhibitors (FK506 and CsA, 10μM each, twice the dose used in Fig 3A and 3B) does not improve the suppression efficiency of PP2B activity which dephosphorylates pS295 induced by the NMDA treatment (20 μM, 15 min) in primary mouse cortical neurons. The data are represented as the mean ± standard deviation overlaid with individual data points (n = 4 from two independent experiments). ***P < 0.001 by two-way ANOVA with the post-hoc Tukey’s multiple comparison test. *F*(1,12) = 184.0, *P* < 0.0001 for FK+CsA, *F*(1,12) = 1019, *P* < 0.0001 for NMDA, and *F*(1,12) = 0.02728, *P* = 0.8716 for interaction.
(TIF)

S1 Data Spread sheet containing quantitative data. (XLSX)

S1 Raw images Original western blot images with membranes stained with Ponceau S. The upper half of the membrane was used to detect pS295 or PSD-95, whereas the lower half was used for pJnk1, Jnk1, pGsk3β, or Gsk3β. Two membranes connected by a dashed line are originally a single membrane at the blotting step. Transferred proteins on the membranes were monitored with Ponceau S staining after the signal detection using chemiluminescence. Some Ponceau-stained membranes (unused half of the membrane) are included as references for monitoring loaded proteins. In the lanes labeled with "E", protein extracts for filling empty lanes are loaded.
(PDF)
