# Supplementary figures and images for: Ca2+-PP2B-PSD-95 axis: A novel regulatory mechanism of the phosphorylation state of Serine 295 of PSD-95
Source: PLoS One. 2024 Nov 7;19(11):e0313441. doi: 10.1371/journal.pone.0313441 (PMC11542788; doi:10.1371/journal.pone.0313441)

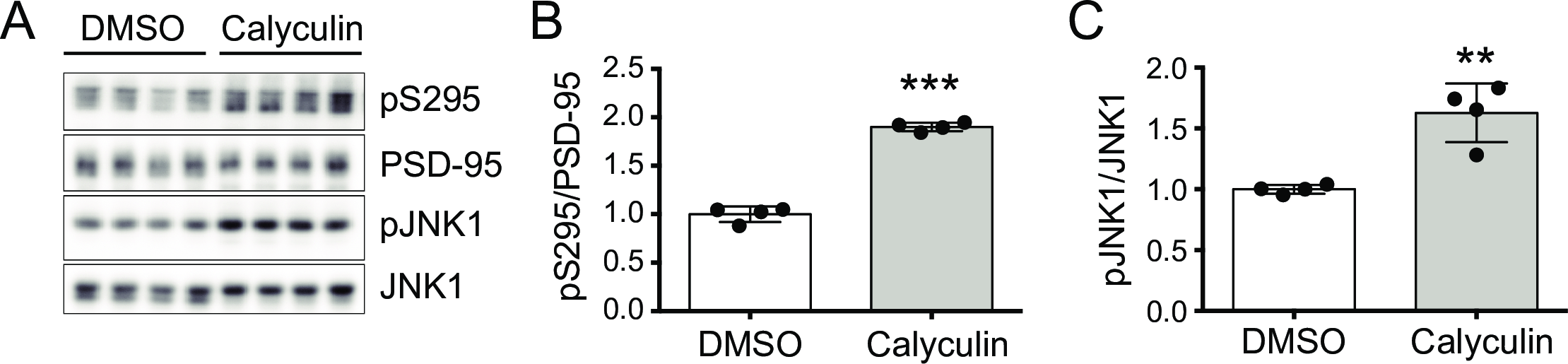

Supplement: S1 Fig — (A) Western-blot analyses of phosphorylated Ser295 of PSD-95 (pS295), total PSD-95, an active form of JNK1 (pJNK1) and total JNK1 in untreated (DMSO) and calyculin A-treated (20 nM, 45 min) primary mouse cortical neurons. (B, C) Quantification of pS295/PSD-95 (B) and pJNK1/JNK1 (C) with the FK506 treatment relative to the untreated condition (DMSO) shown in (A). The data are represented as the mean ± standard deviation overlaid with individual data points (n = 4). ***P < 0.001, **P < 0.01 by the unpaired Student’s t-test; n.s., not significant. (TIF) [file pone.0313441.s001.tif]

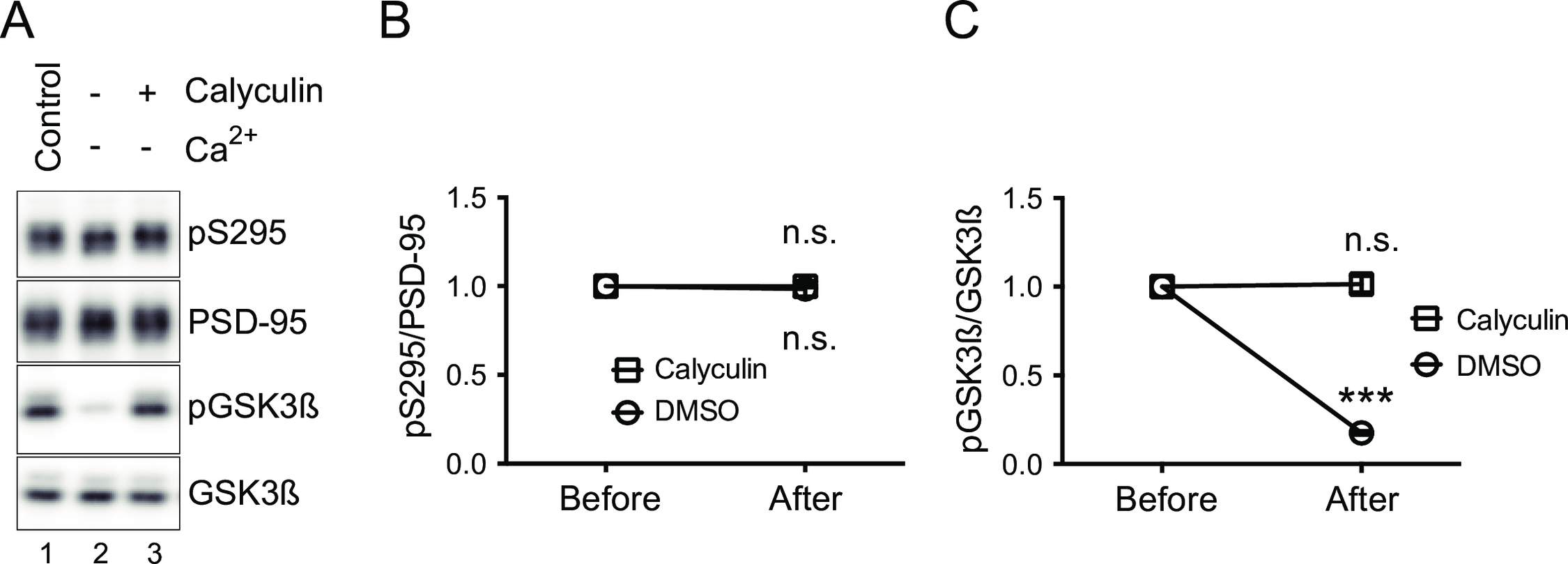

Supplement: S2 Fig — (A) Western-blot analysis showing that PP1/PP2A was active in our in-vitro dephosphorylation assay. The decrease of phosphorylated GSK3β (pGSK3β(S9)) was completely suppressed by the PP1/PP2A inhibitor calyculin A. (B, C) Quantification of pS295/PSD-95 (B) and pGSK3/β/GSK3β (C) incubated with or without calyculin A relative to the no-incubation (Control) condition. The data are represented as the mean ± standard deviation (n = 3 from three independent experiments). ***P < 0.001 by the unpaired Student’s t-test; n.s., not significant. (TIF) [file pone.0313441.s002.tif]

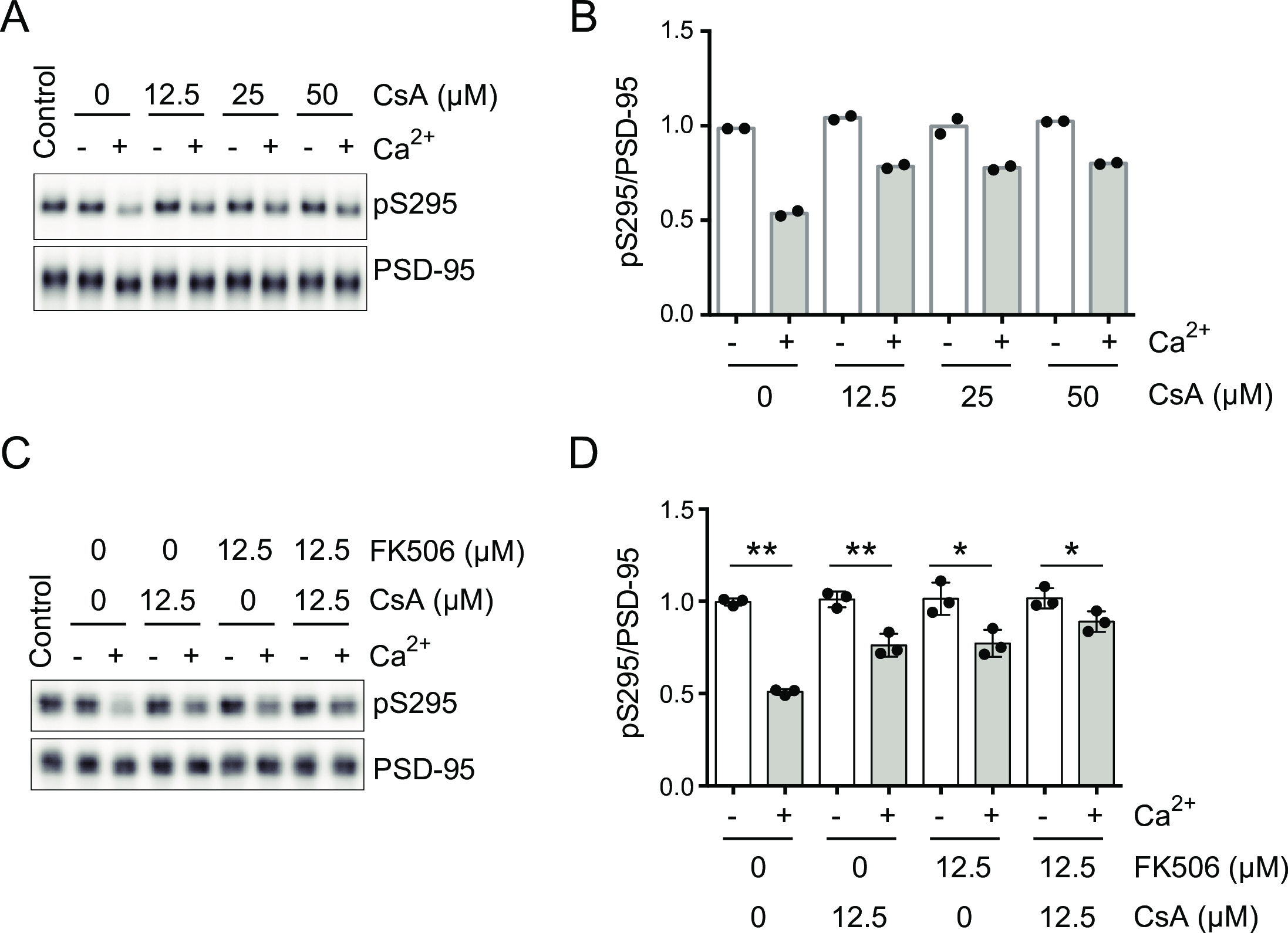

Supplement: S3 Fig — (A, B) Western-blot analysis (A) and quantification (B) of the efficiency of suppression by different doses of CsA on the Ca2+-dependent dephosphorylation of pS295 in vitro. Bars show the mean value and individual data points are shown as black dots (n = 2 from two independent experiments). (C, D) Western-blot analysis (C) and quantification (D) showing the incomplete suppression of the Ca2+-dependent dephosphorylation of pS295 in vitro by coapplication of FK506 and CsA. The data are represented as the mean ± standard deviation overlaid with individual data points (n = 3 from three independent experiments). **P < 0.01, *P < 0.05 by the unpaired Student’s t-test. (TIF) [file pone.0313441.s003.tif]

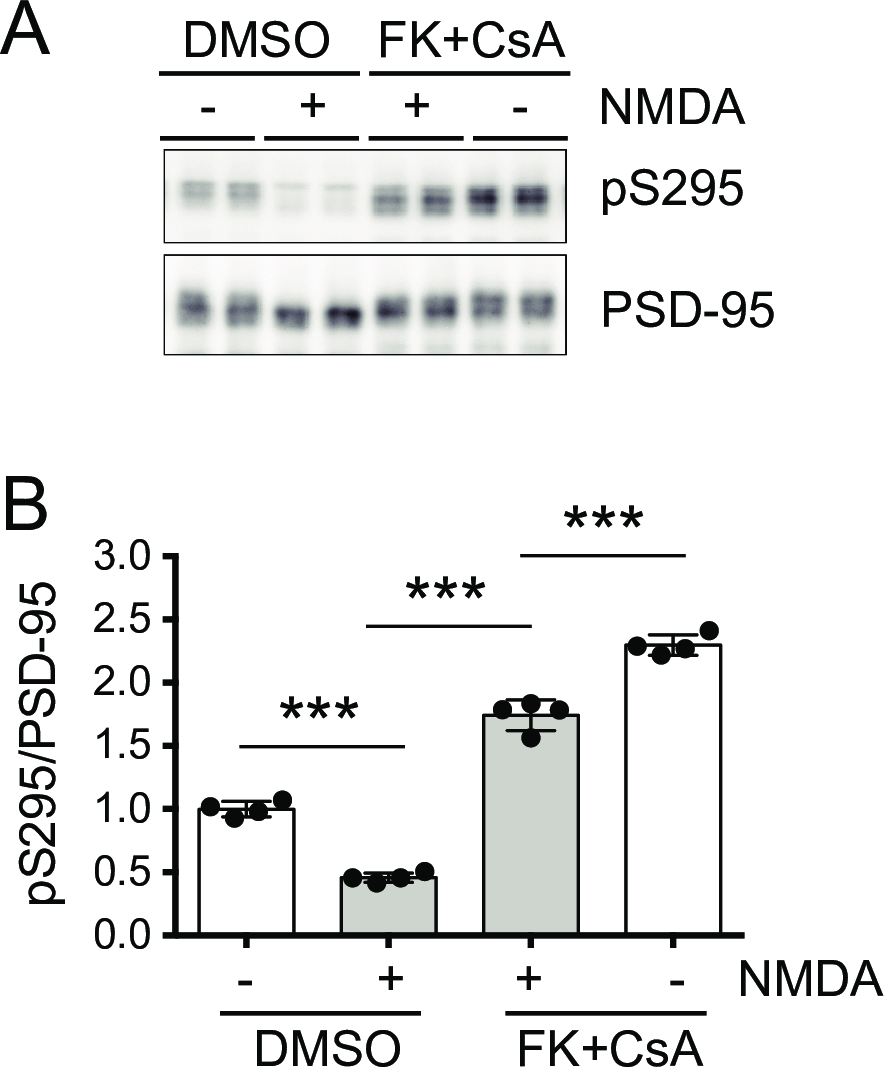

Supplement: S4 Fig — (A, B) Western-blot analysis (A) and quantification (B) showing that an increased dose of the mixture of PP2B inhibitors (FK506 and CsA, 10μM each, twice the dose used in Fig 3A and 3B) does not improve the suppression efficiency of PP2B activity which dephosphorylates pS295 induced by the NMDA treatment (20 μM, 15 min) in primary mouse cortical neurons. The data are represented as the mean ± standard deviation overlaid with individual data points (n = 4 from two independent experiments). ***P < 0.001 by two-way ANOVA with the post-hoc Tukey’s multiple comparison test. F(1,12) = 184.0, P < 0.0001 for FK+CsA, F(1,12) = 1019, P < 0.0001 for NMDA, and F(1,12) = 0.02728, P = 0.8716 for interaction. (TIF) [file pone.0313441.s004.tif]
