# Supplementary material for: Ca2+-PP2B-PSD-95 axis: A novel regulatory mechanism of the phosphorylation state of Serine 295 of PSD-95
Source: PLoS One. 2024 Nov 7;19(11):e0313441. doi: 10.1371/journal.pone.0313441 (PMC11542788; doi:10.1371/journal.pone.0313441)
Supplement: S1 Raw images — The upper half of the membrane was used to detect pS295 or PSD-95, whereas the lower half was used for pJnk1, Jnk1, pGsk3β, or Gsk3β. Two membranes connected by a dashed line are originally a single membrane at the blotting step. Transferred proteins on the membranes were monitored with Ponceau S staining after the signal detection using chemiluminescence. Some Ponceau-stained membranes (unused half of the membrane) are included as references for monitoring loaded proteins. In the lanes labeled with "E", protein extracts for filling empty lanes are loaded. (PDF) [file pone.0313441.s006.pdf]

Original WB images (Fig 1A)

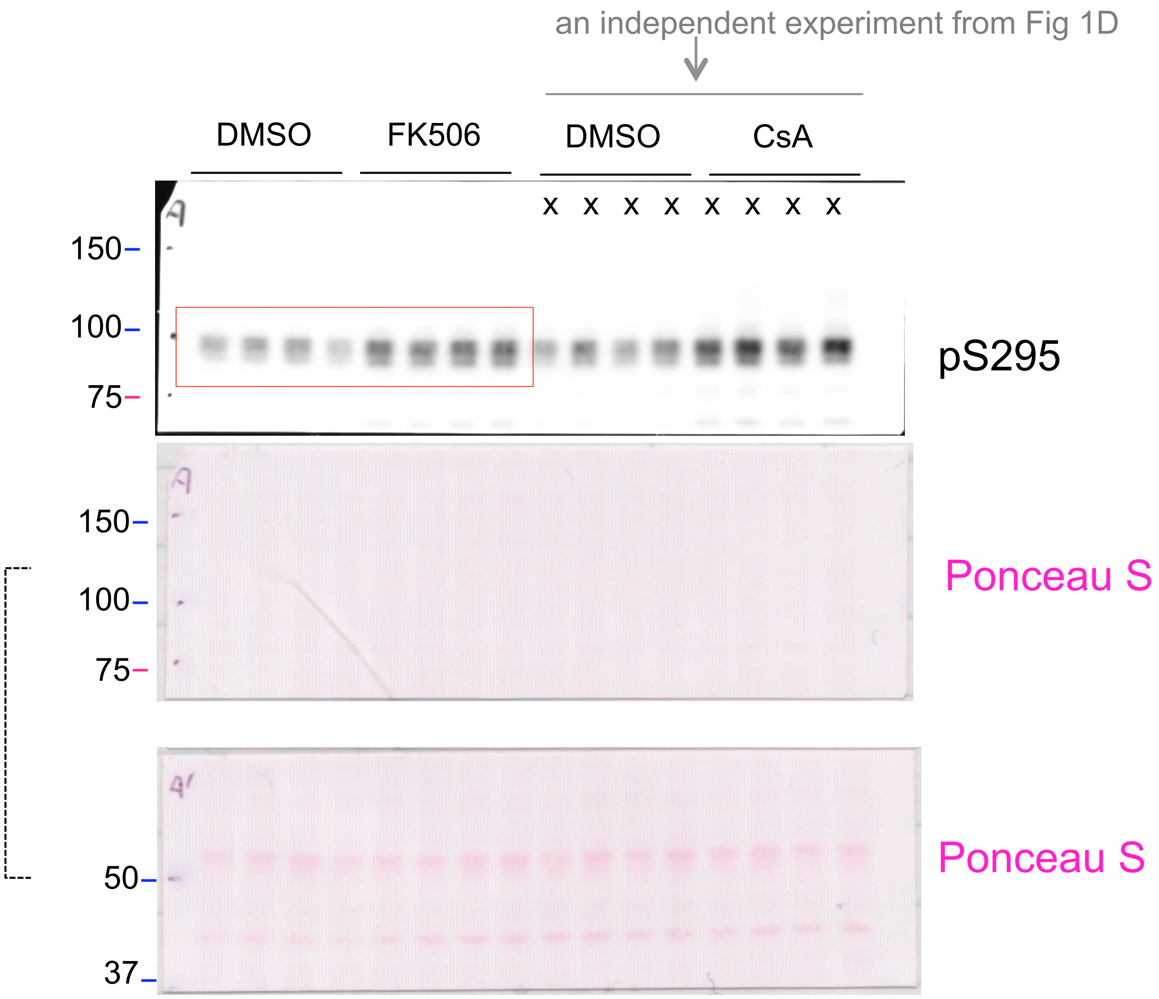

lower half of the membrane not used for pS295 detection

Original WB images (Fig 1A)

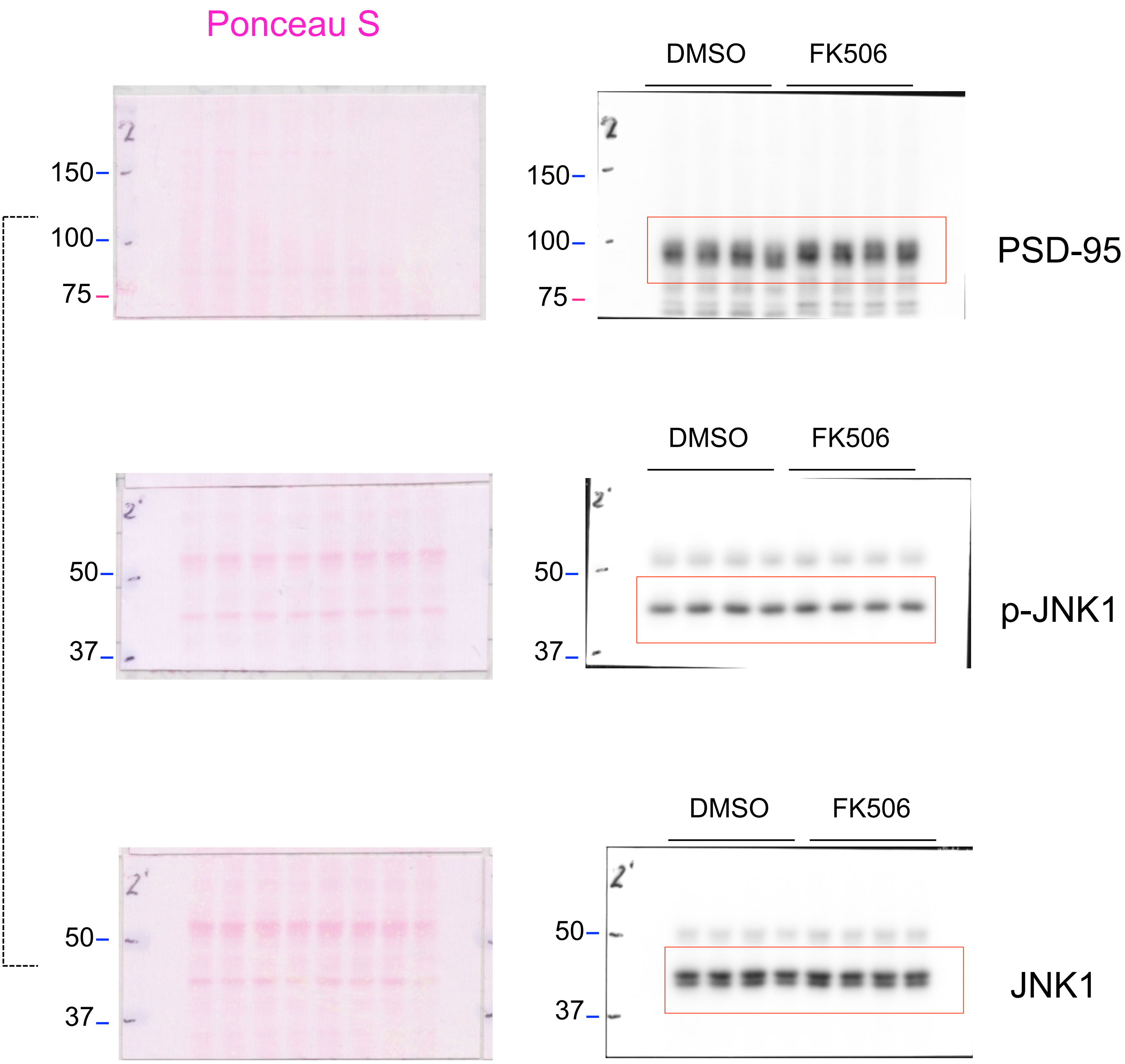

Original WB images (Fig 1D)

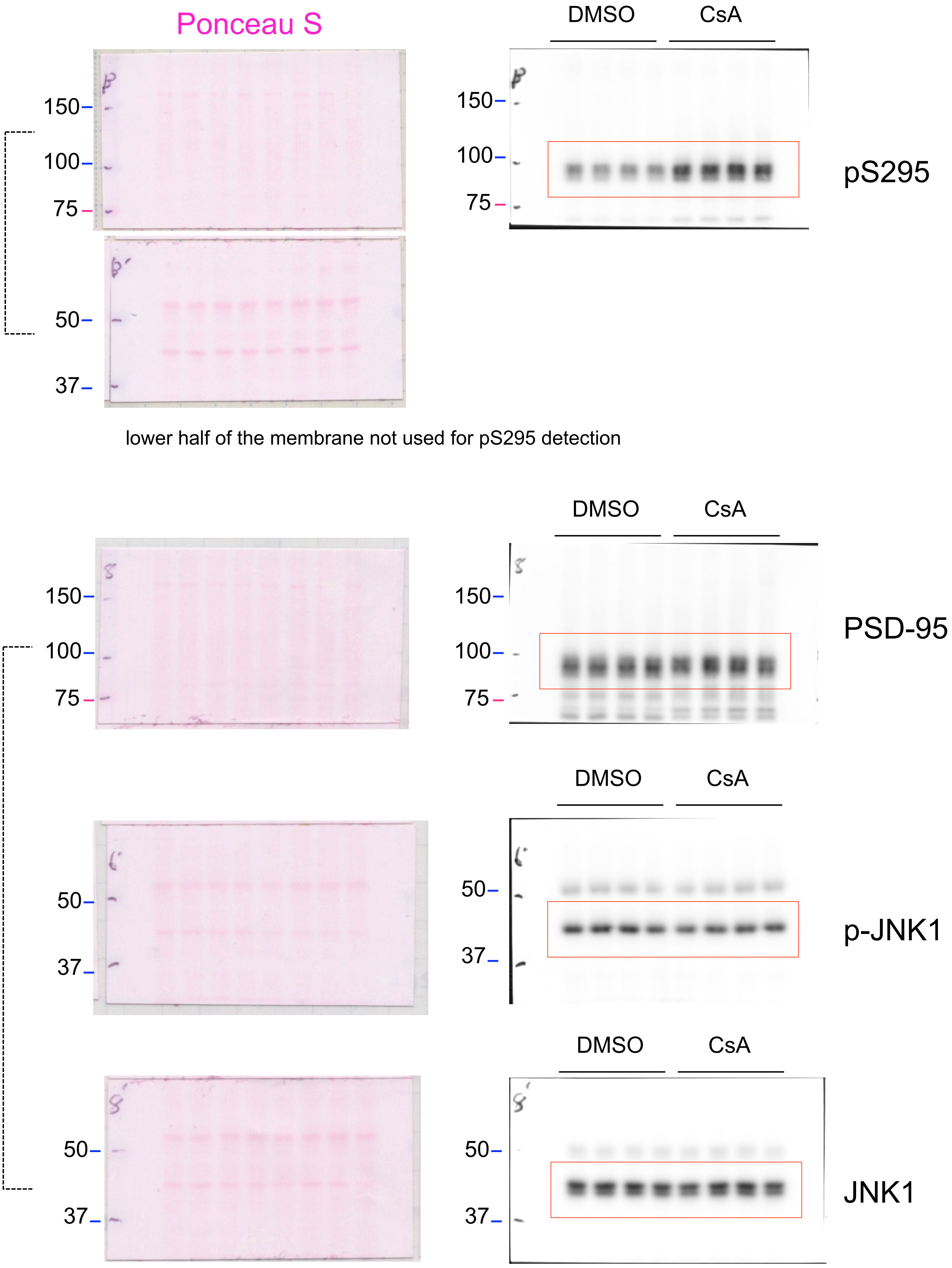

Original WB images (Fig 1G)

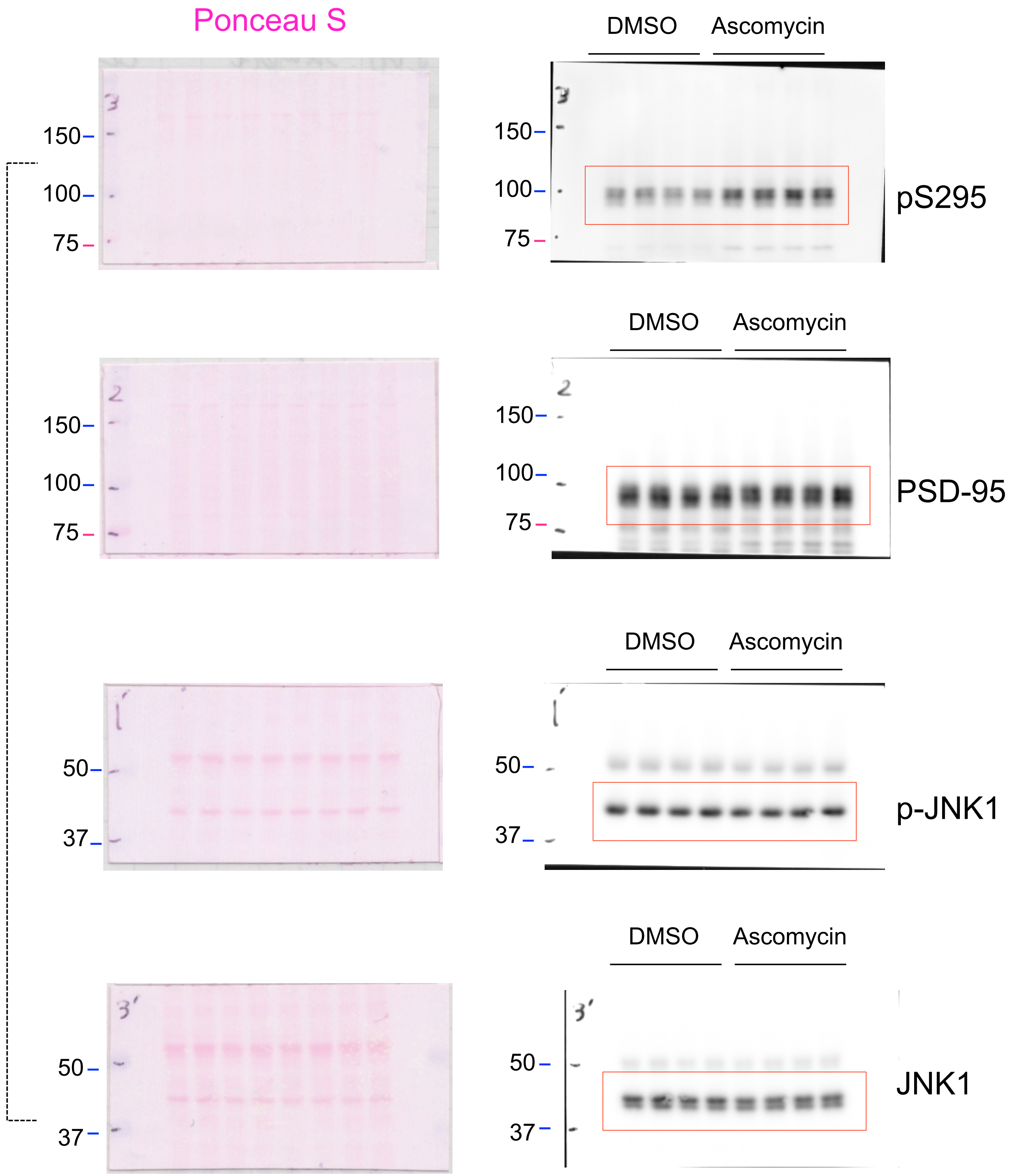

Original WB images (Fig 2A)

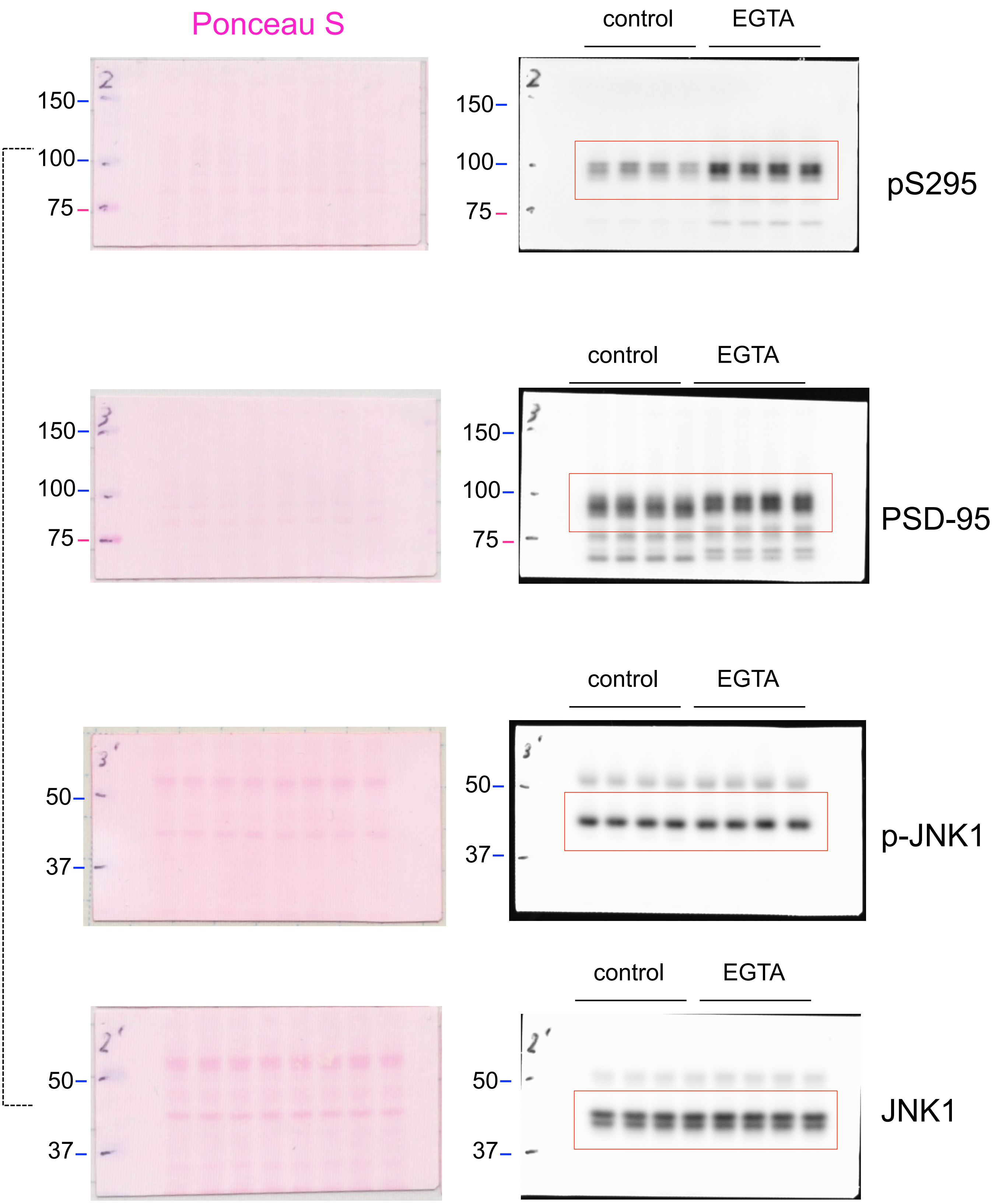

Original WB images (Fig 2D)

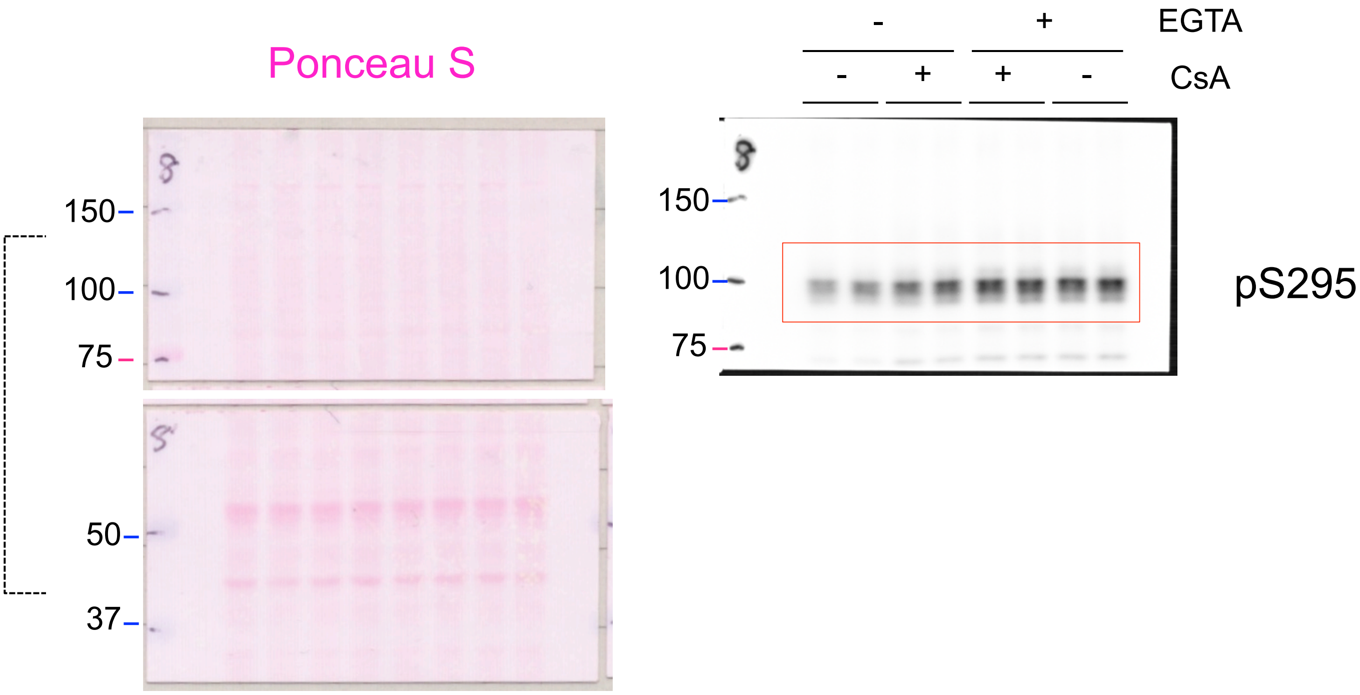

lower half of the membrane not used for pS295 detection

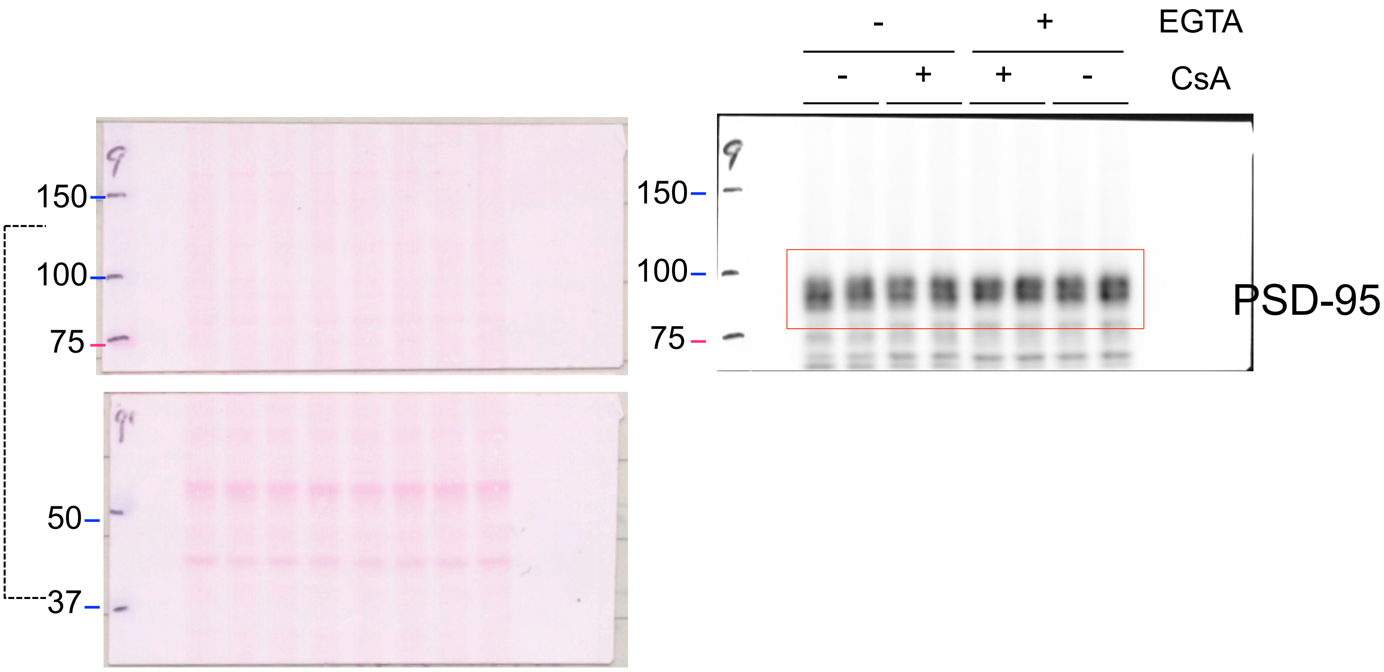

lower half of the membrane not used for PSD-95 detection

Original WB images (Figs 3A and 3C)

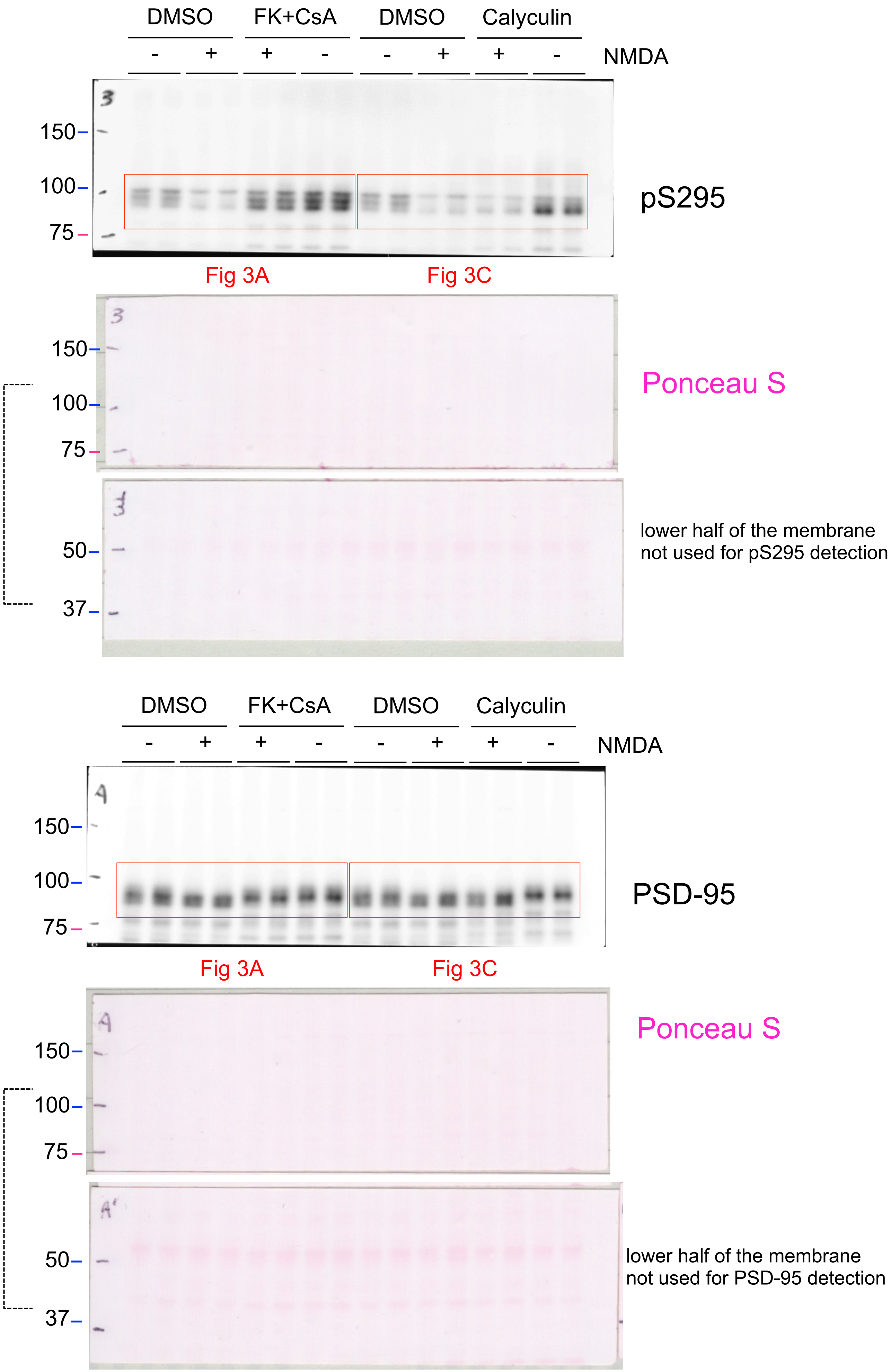

Original WB images (Fig 4A)

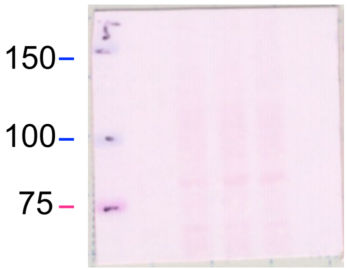

Ponceau S

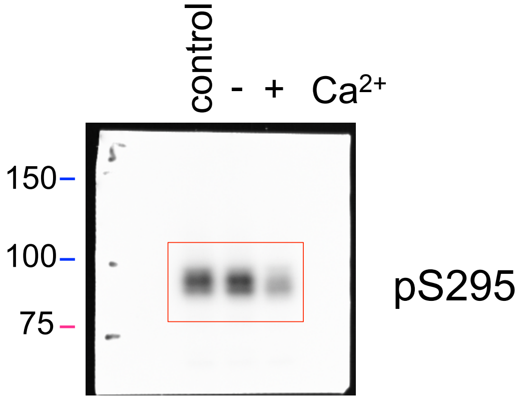

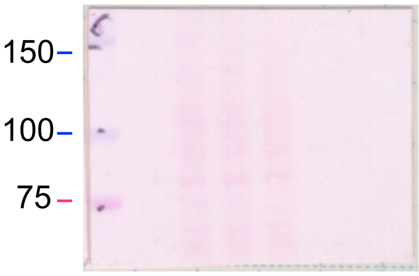

Ponceau S

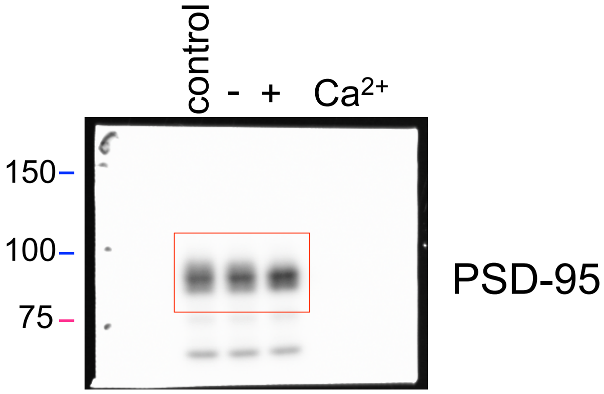

Original WB images (Fig 4C)

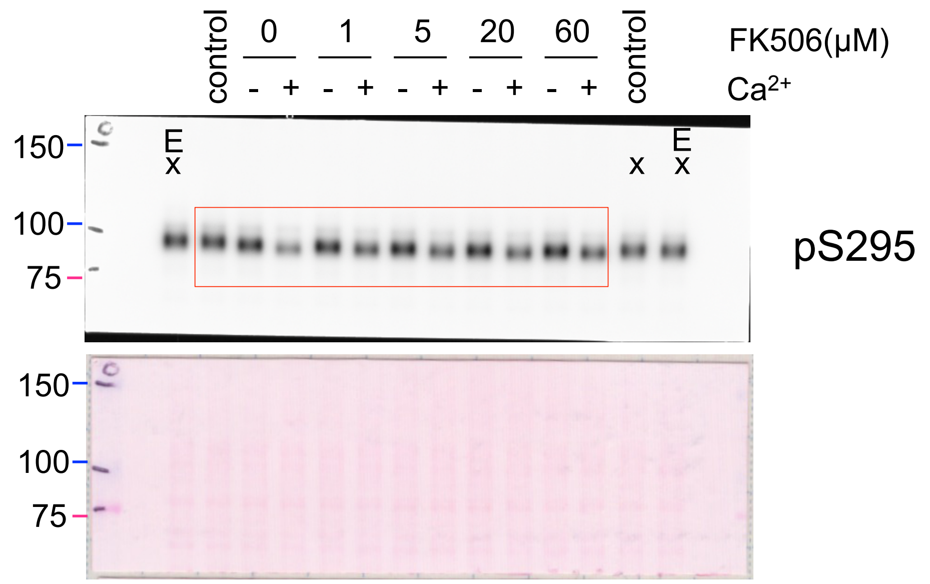

Ponceau S

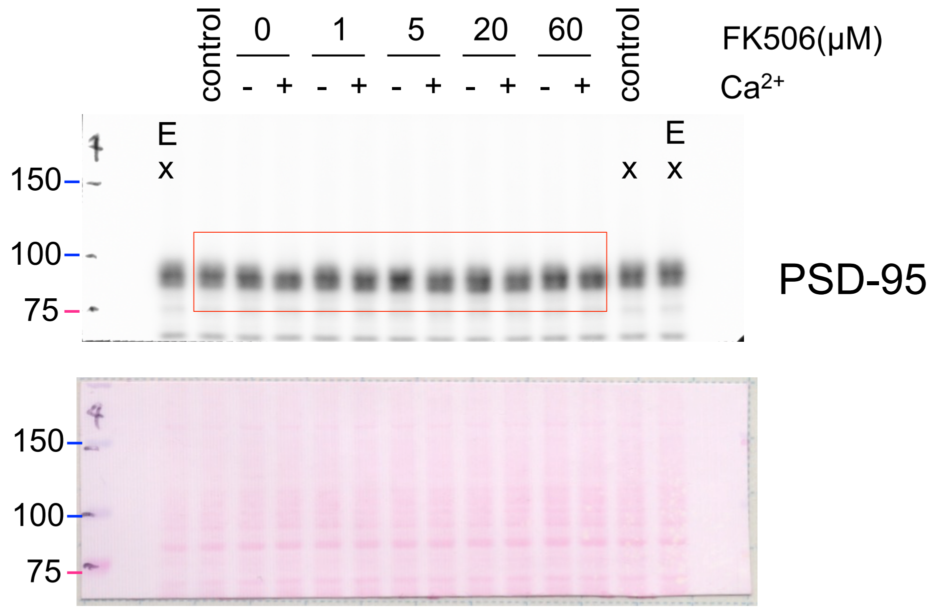

Ponceau S

Original WB images (Fig 4E)

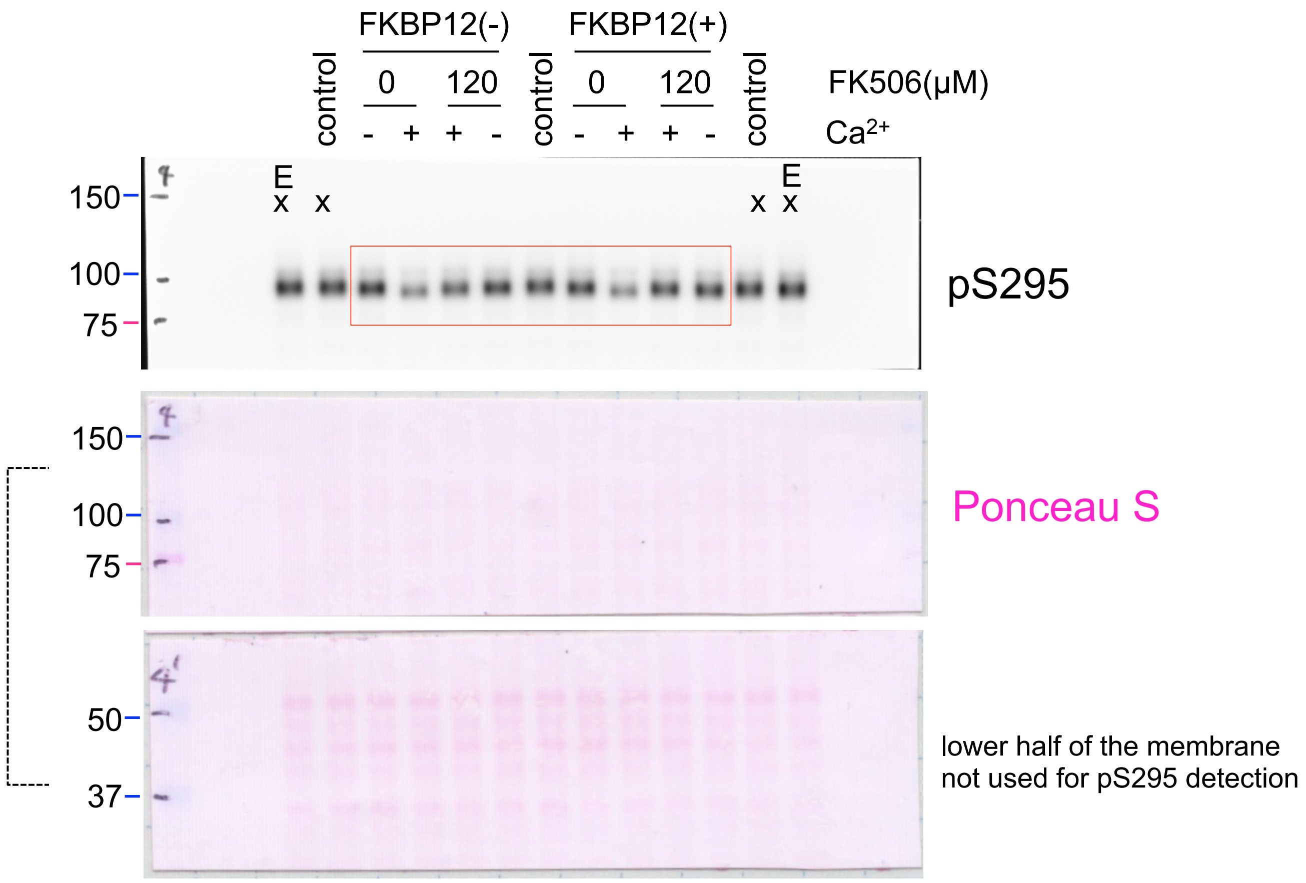

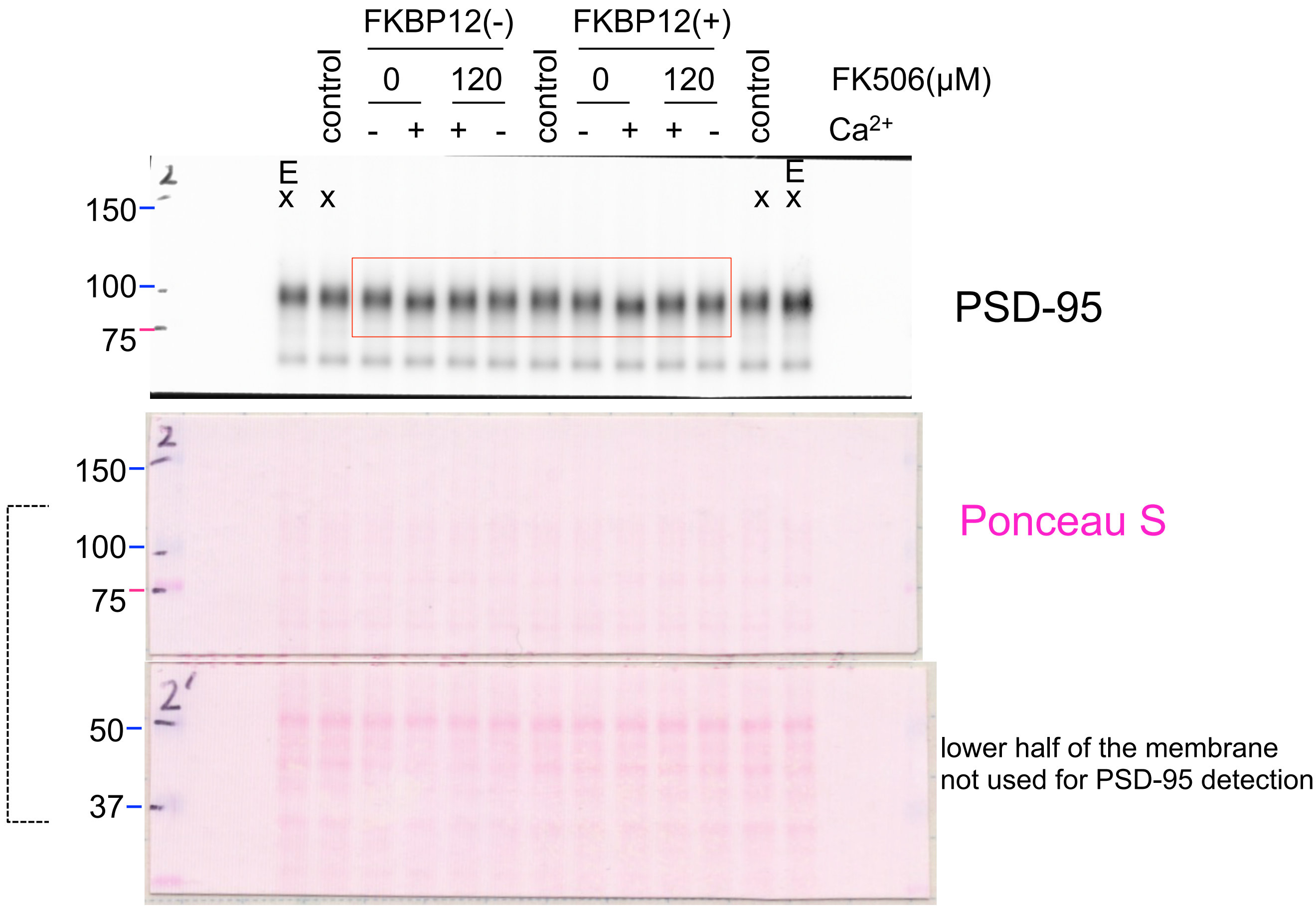

Original WB images (S1A Fig)

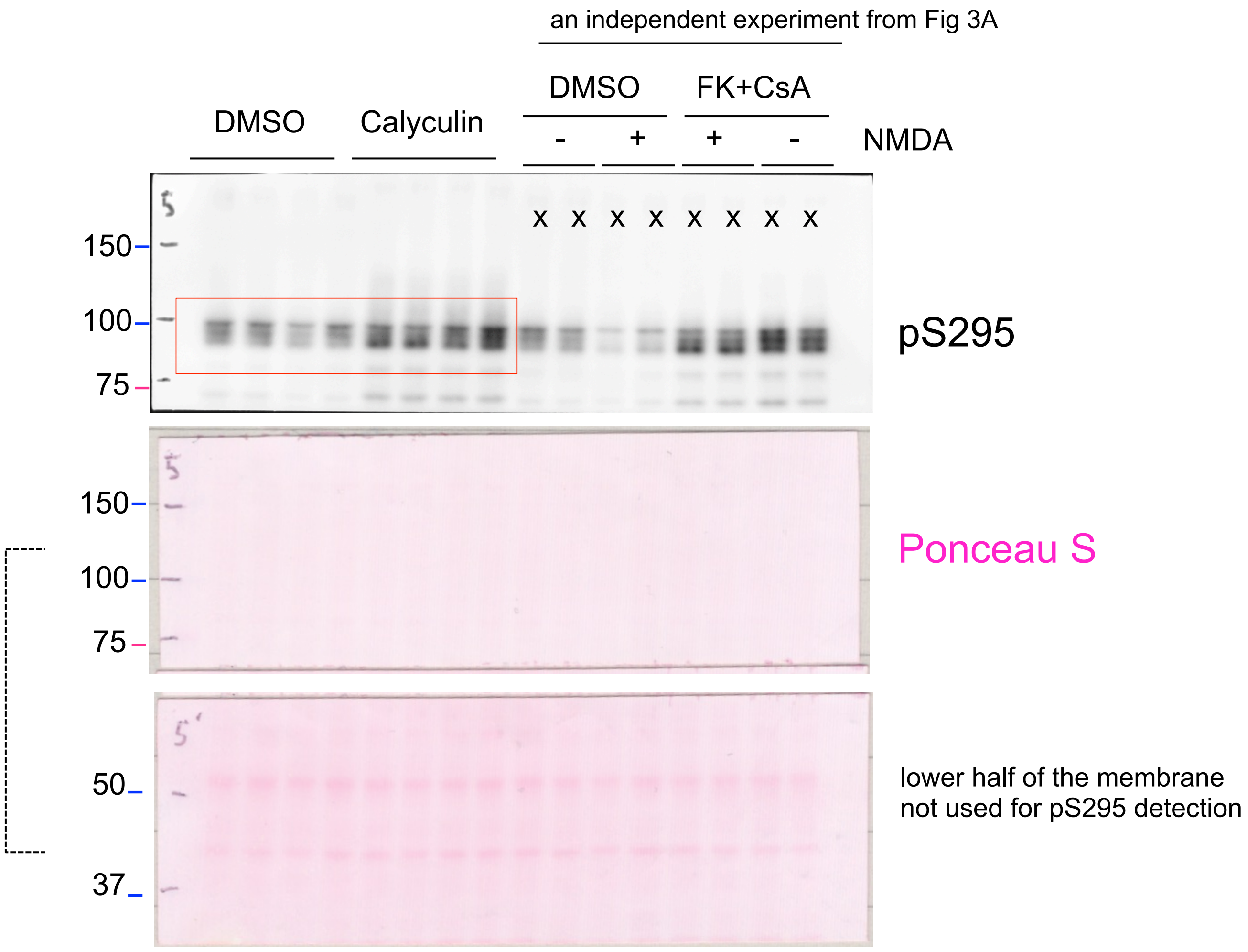

Original WB images (S1A Fig)

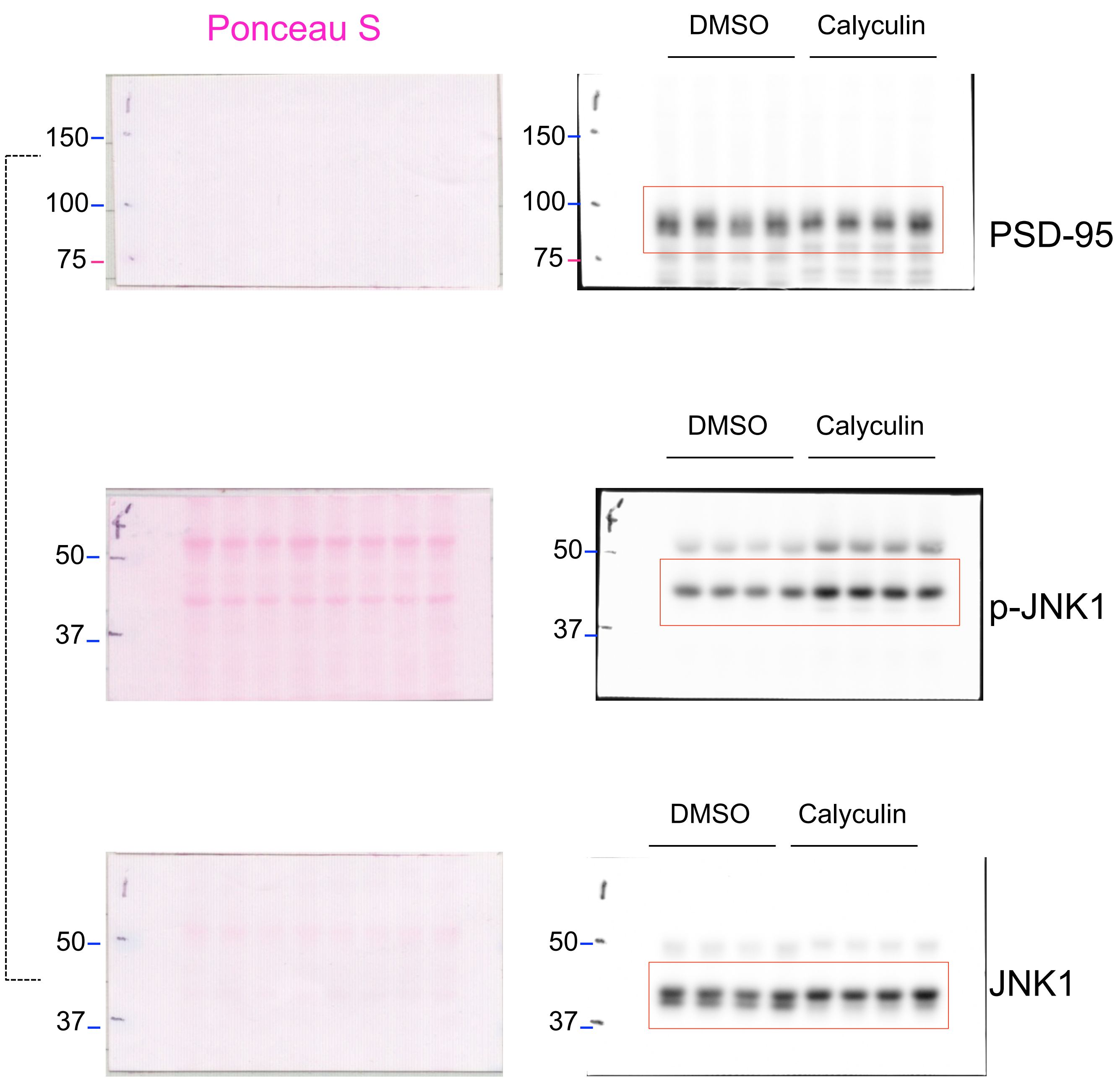

Original WB images (S2A Fig)

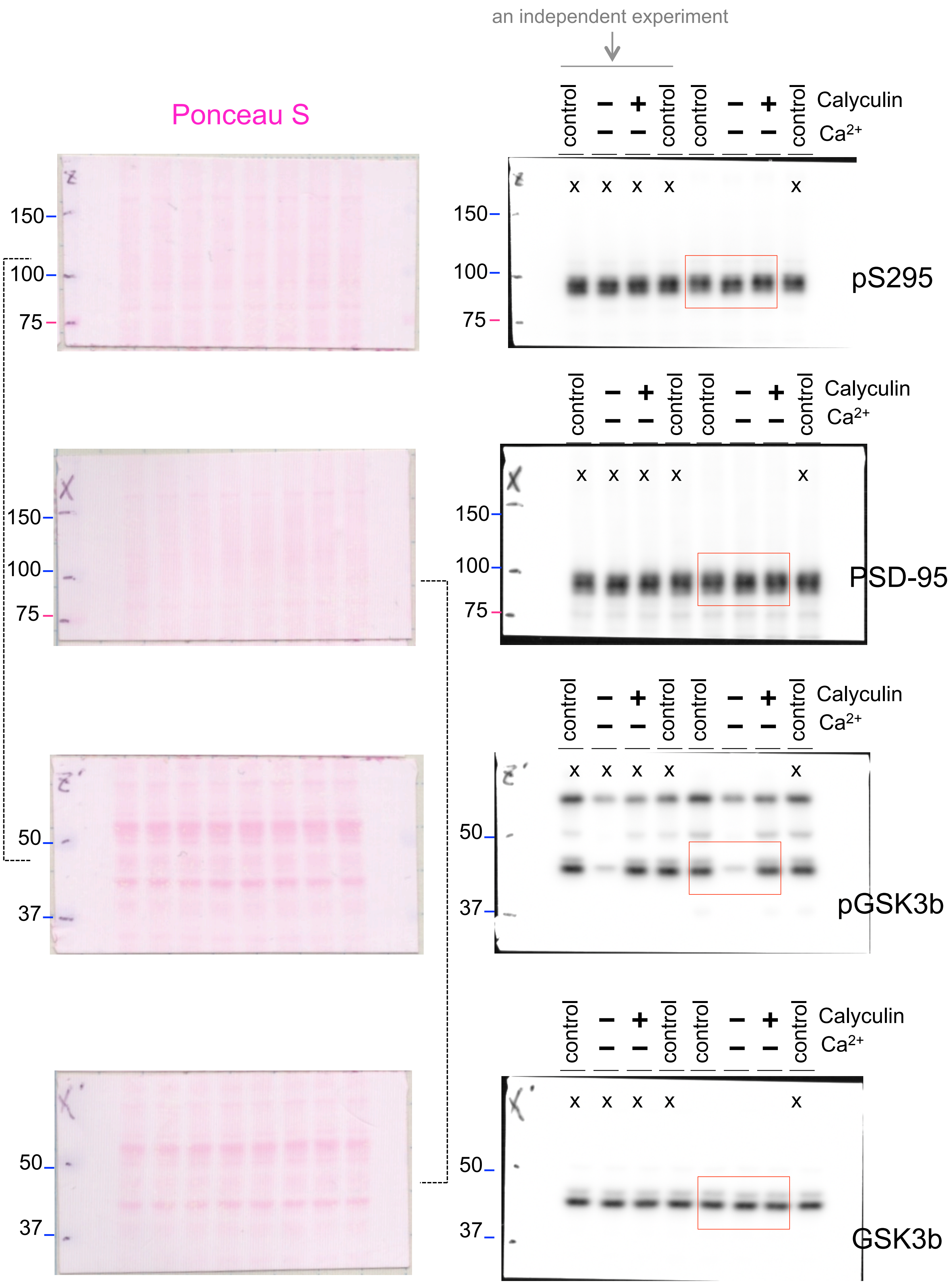

Original WB images (S3A Fig)

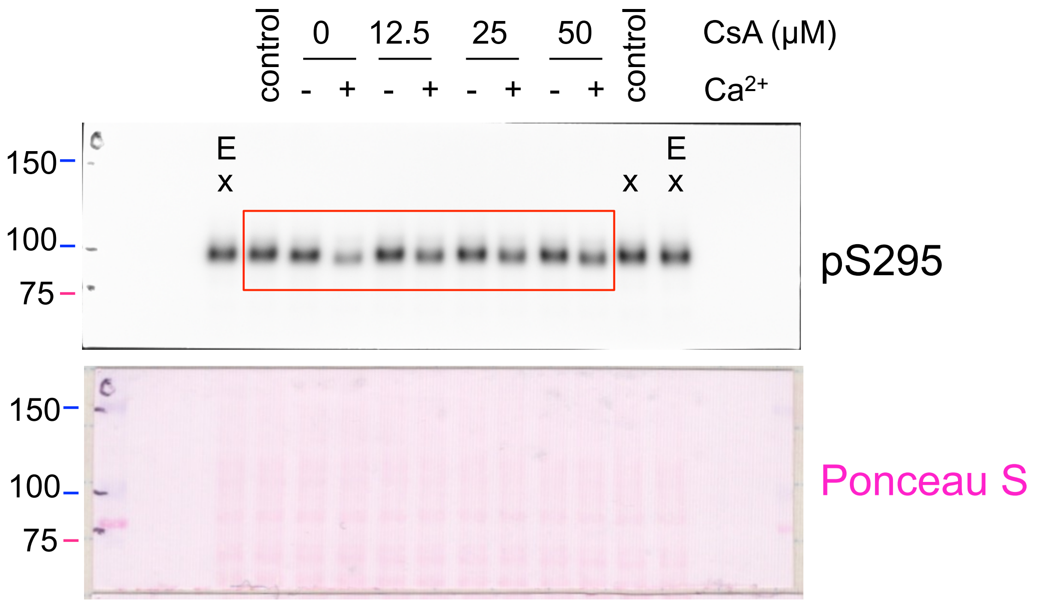

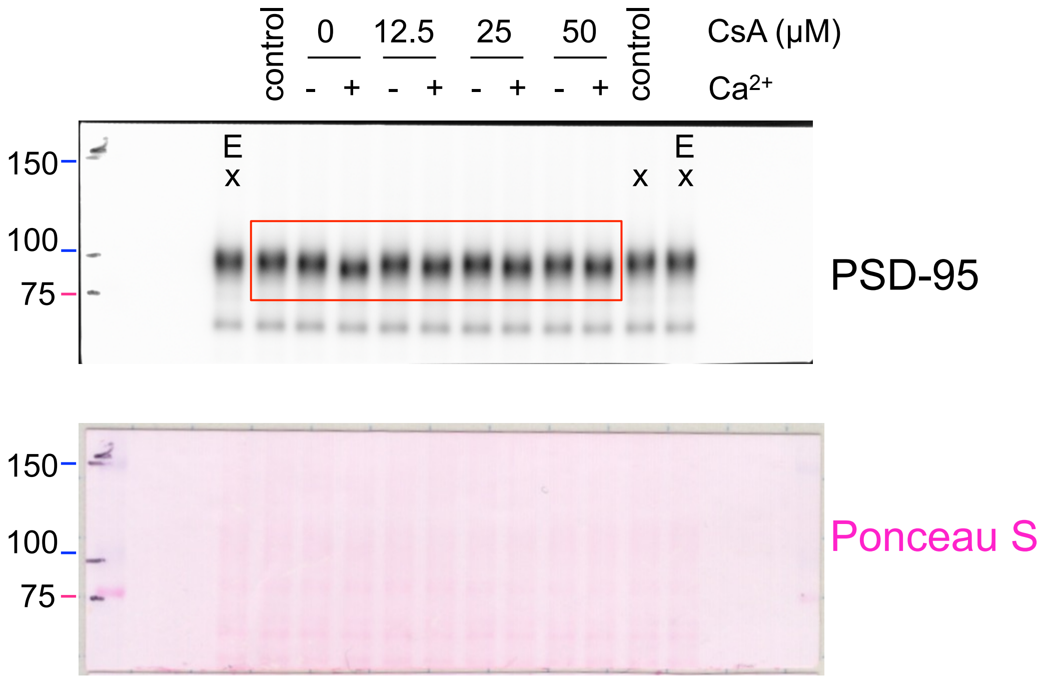

Original WB images (S3C Fig)

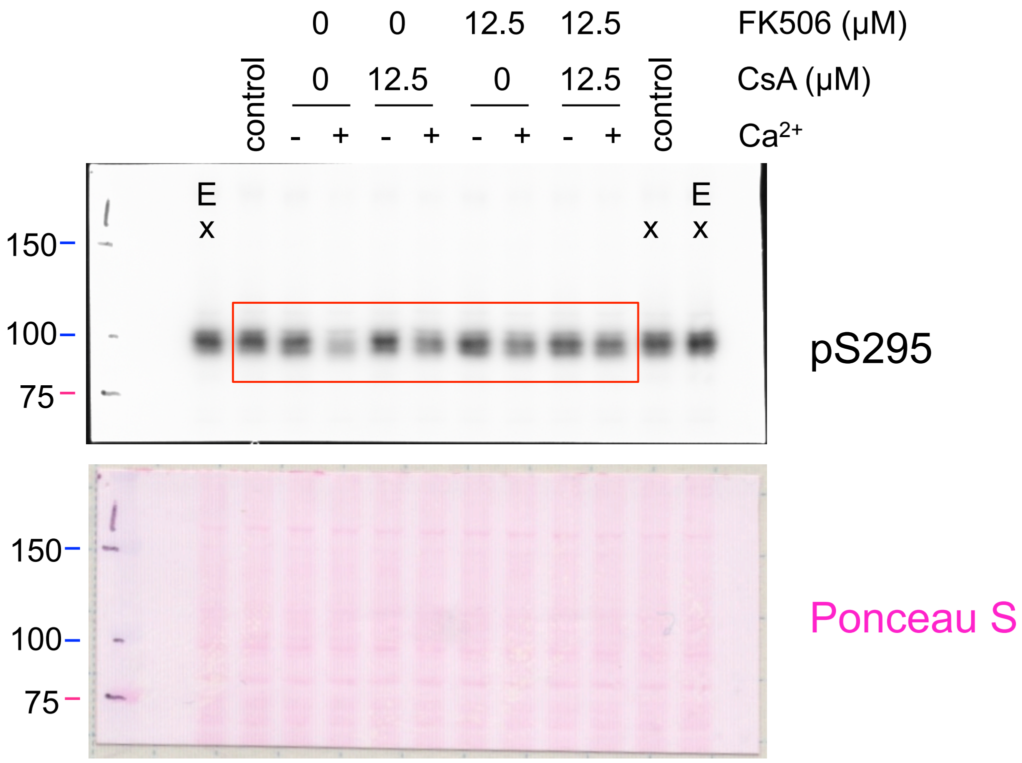

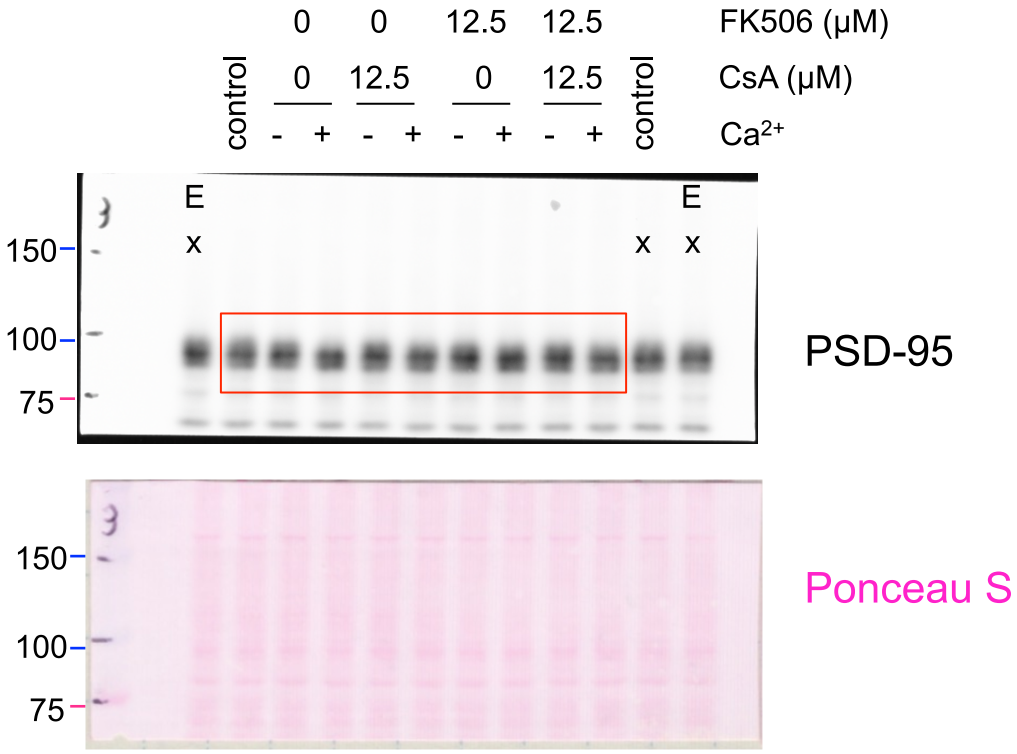

Original WB images (S4A Fig)

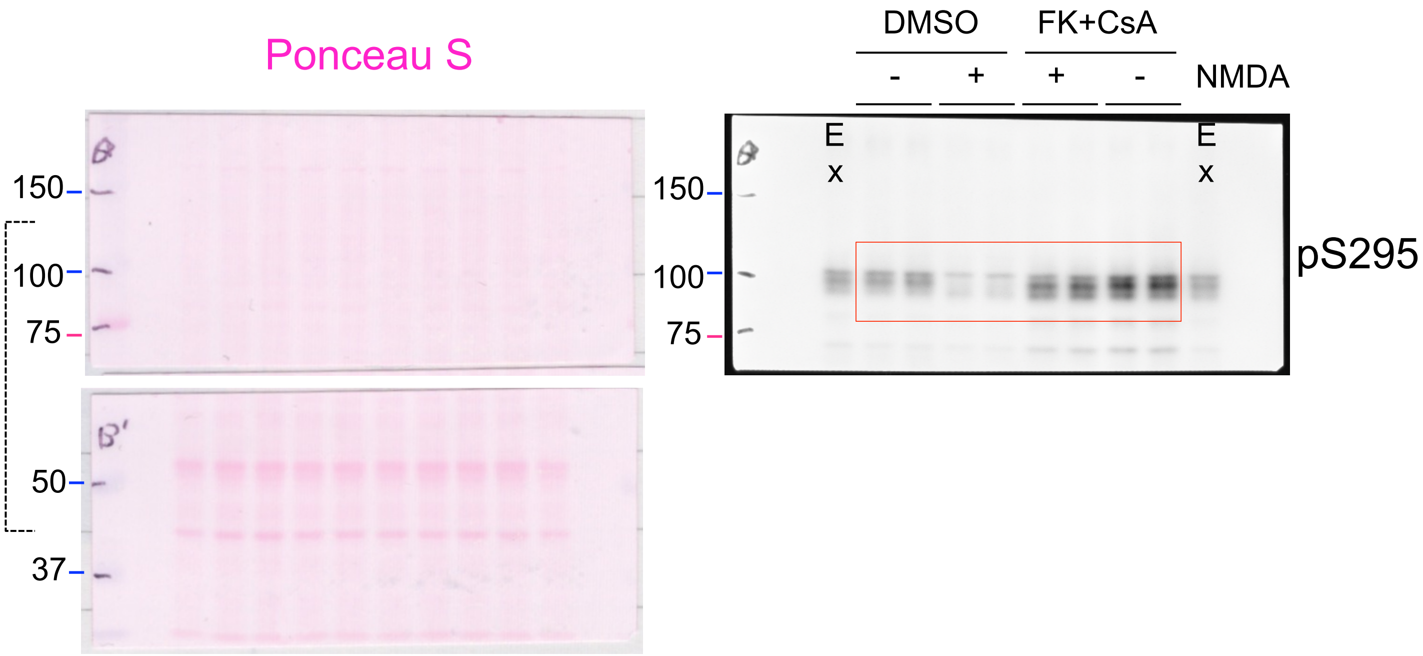

lower half of the membrane
not used for pS295 detection

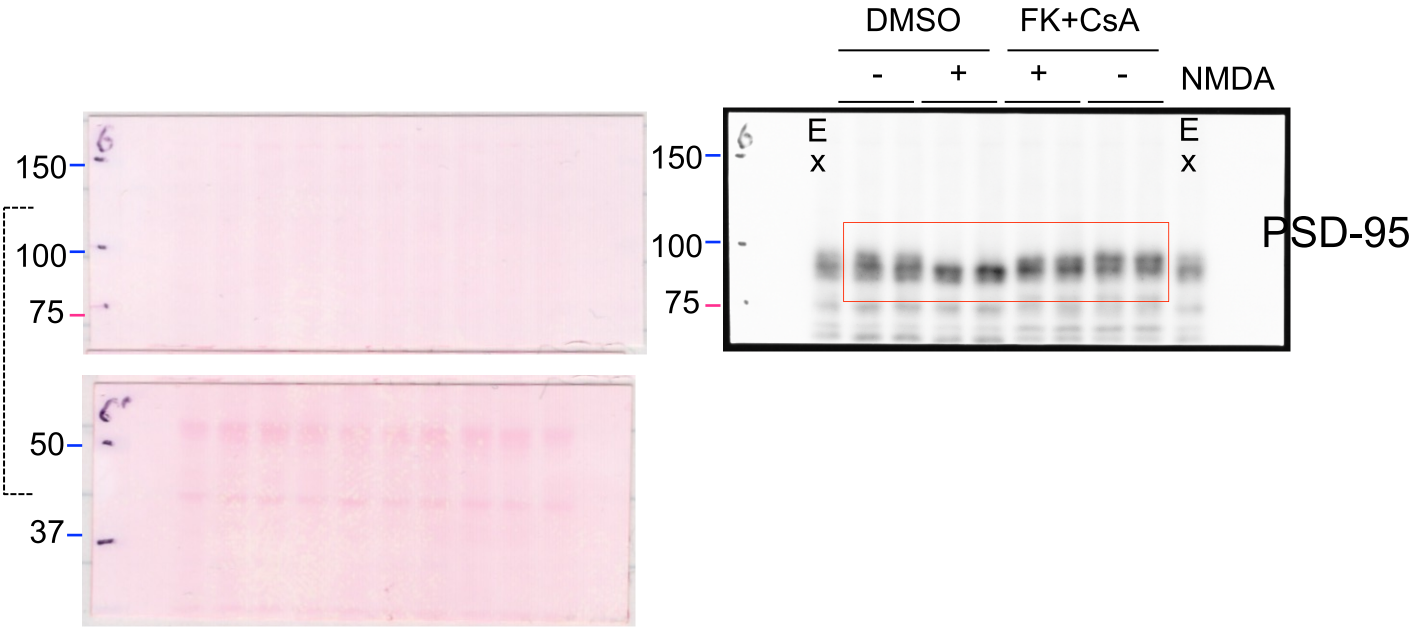

lower half of the membrane not
used for PSD-95 detection
